# Chondrogenic primed extracellular vesicles activate miR-455/SOX11/FOXO axis for cartilage regeneration and osteoarthritis treatment

**DOI:** 10.1038/s41536-022-00250-7

**Published:** 2022-09-16

**Authors:** Ye Sun, Jie Zhao, Qiang Wu, Yuxin Zhang, Yongqing You, Wenbo Jiang, Kerong Dai

**Affiliations:** 1 Department of Orthopaedics, The First Affiliated Hospital of Nanjing Medical University, Nanjing, Jiangsu 210029 China; 2 Clinical and Translational Research Center for 3D Printing Technology, Shanghai Key Laboratory of Orthopaedic Implants, Department of Orthopaedic Surgery, Shanghai Ninth People’s Hospital, Shanghai Jiao Tong University School of Medicine, Shanghai, 200011 China; 3 Gastrointestinal Surgery and Central Laboratory, The Affiliated Changzhou No. 2 People’s Hospital of Nanjing Medical University, 68 Gehu Road, Changzhou, Jiangsu 213003 China; 4 Department of Rehabilitation Medicine, Shanghai Ninth People’s Hospital Affiliated to Shanghai Jiao Tong University School of Medicine, Shanghai, 200011 People’s Republic of China; 5 Department of Nephrology, Affiliated Hospital of Nanjing Medical University, North District of Suzhou Municipal Hospital, Suzhou, China

**Keywords:** Osteoarthritis, Regenerative medicine

## Abstract

Osteoarthritis (OA) is the leading cause of disability worldwide. Considerable progress has been made using stem-cell-derived therapy. Increasing evidence has demonstrated that the therapeutic effects of BMSCs in chondrogenesis could be attributed to the secreted small extracellular vesicles (sEVs). Herein, we investigated the feasibility of applying engineered EVs with chondrogenic priming as a biomimetic tool in chondrogenesis. We demonstrated that EVs derived from TGFβ3-preconditioned BMSCs presented enriched specific miRNAs that could be transferred to native BMSCs to promote chondrogenesis. In addition, We found that EVs derived from TGFβ3-preconditioned BMSCs rich in miR-455 promoted OA alleviation and cartilage regeneration by activating the SOX11/FOXO signaling pathway. Moreover, the designed T3-EV hydrogel showed great potential in cartilage defect treatment. Our findings provide new means to apply biosafe engineered EVs from chondrogenic primed-BMSCs for cartilage repair and OA treatment, expanding the understanding of chondrogenesis and OA development modulated by EV-miRNAs in vivo.

## Introduction

Degenerative joint disease is the leading cause of disability worldwide^1^. Cartilage defects are common in OA development and have become a tricky clinical problem due to the limited self-healing capacity caused by its low cellularity and avascular nature. Major efforts, such as using bone marrow mesenchymal stem cells (BMSCs), have been made in hopes of achieving cartilage repair or OA treatment, which shed light on solving the medical dilemma through regenerative strategies^2,3^. The potential applications of BMSCs for cartilage regeneration and OA treatment have been extensively studied before^4–9^. BMSCs show pluripotency in differentiating into many cell types. OA therapy with Intra-articular transplanted BMSCs has shown potential in facilitating cartilage regeneration with chondrogenic differentiation capabilities, anti-inflammatory effects, and immunomodulatory properties^10–12^. Our previous research also indicated that TGFβ3-priming of BMSCs could generate hyaline cartilage on the articular surface, and chondrogenic differentiation of BMSCs is a complicated process induced by complex hormonal cocktails in vitro^13^. However, these classical chondrogenic cocktails have not yet been applied in clinical trials, besides, they’re far from satisfactory due to their chondrogenic inefficiency and potential oncogenicity in vivo. Moreover, neither is it possible to replicate the natural chondrogenic process by these cocktails in vivo^14^. In spite of such difficulties, increasing evidence has demonstrated that the therapeutic effects of BMSCs in chondrogenesis could be a solution thanks to the paracrine factors secreted by BMSCs, particularly small extracellular vesicles (sEVs)^4,15^. It’s known that the application of sEVs is the potential in initiating cartilage regeneration for their properties of transporting miRNAs and building cellular interactions. Specifically, under the modification of miRNA, BMSCs are able to stably differentiate into chondrocytes and preserve relevant phenotypes, thus having gained much attention in regenerative research recently. Based on previous studies, BMSC-derived EVs have biological functions similar to BMSCs, and play an indispensable role in suppressing inflammation, regulating immune responses, and repairing tissue damage^6,16–18^. In this way, bioactive molecules contained within EVs could be modified by modulating the state of the donor cells, which makes it possible to engineer EVs derived from preconditioned BMSCs for targeted tissue regeneration in vivo^4,19^. On the other side, many cytokines have proven their functions in regulating chondrogenesis, such as Wnt16 and Interleukin-1β, which indicates the feasible way of engineering EVs with certain cytokines to achieve cartilage regeneration. (3, 4) Amongst all, TGFβ3 has shown efficacy to promote chondrogenic differentiation of BMSCs to form hyaline cartilage in many previous studies^12,13^. As a consequence, it’s reasonable to assume that the resulting EVs in TGFβ3 treatment could trigger stable chondrogenesis, and this assumption is worthy of further elaboration.

**Fig. 1:**
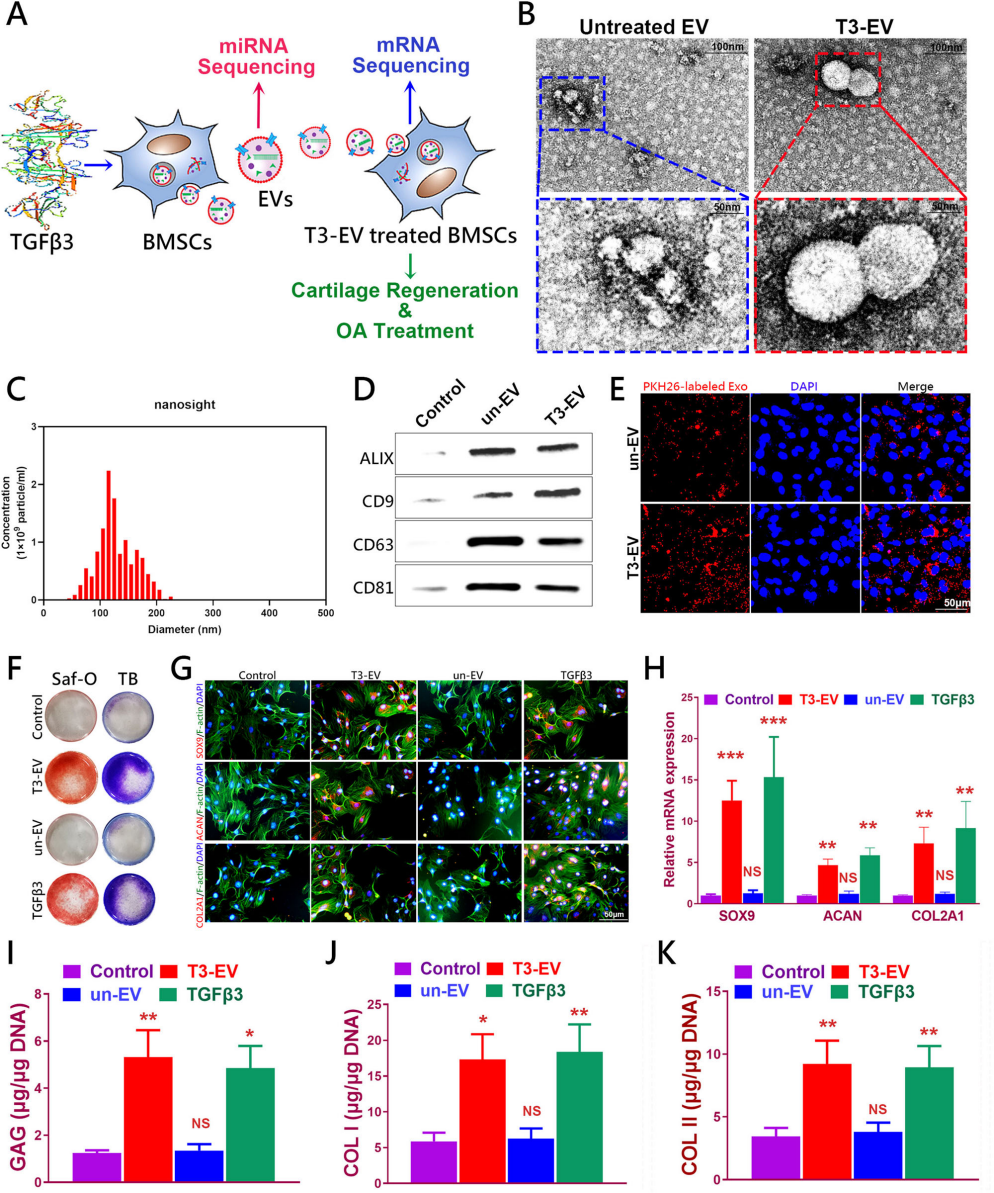
Identification of T3-EV and un-EV. **A** Illustration of study design. **B** Representative images showing the morphology of T3-EV and un-EV visualized under transmission electron microscopy (TEM). **C** Particle size distribution of h T3-EVs measured using nanoparticle tracking Analysis (NTA). **D** Quantification of surface markers of EVs evaluated by western blotting. BMSCs served as the control in the western blot analysis of surface markers of EVs. **E** Representative fluorescence micrograph of PKH26 (red)-labeled EVs internalized by primary BMSCs. The labeled EVs were co-incubated with BMSCs for 24 h. **F** GAG staining with Safranin-O and Toluidine blue staining of BMSCs treated with EVs for 21 days. **G** Chondrogenesis was defined with immunostaining of SOX9, ACAN, and COL2A1(red). Counterstaining with F-actin (green) and DAPI (blue) was applied. **H** Three different experiments with the same BMSCs and the same EVs were performed. Chondrogenic gene expression (*n* = 3 for each) was assayed with qRT-PCR for SOX9, ACAN, and COL2A1. **I–K** Quantification of deposited GAGs and collagens (*n* = 3 for each) was also performed with the same BMSCs and the same EVs to demonstrate the chondrogenic lineage committed by the BMSCs. Treatment with saline served as control. *P < 0.05, **P < 0.01, ***P < 0.001, NS not significant.

In the present study, we evaluated the feasibility of applying engineered EVs as a biomimetic tool in chondrogenesis. We demonstrated that when BMSCs were preconditioned by TGFβ3, and EVs secreted from these BMSCs could then present with enriched specific chondrogenic miRNAs, which could act on native cells and eventually contribute to chondrogenesis. In addition, miR-455, one of many highly enriched miRNAs contained by the EVs-, targets SOX11 and further regulates the downstream FOXO signaling pathway to enhance chondrogenesis. Taken together, our findings provide new means to apply biosafe engineered EVs from chondrogenic preconditioned-BMSCs for cartilage repair and OA treatment, expanding understandings of chondrogenesis and OA development modulated by EV-miRNAs in vivo. The diagram of our design is generally demonstrated in Fig. 1A.

## Results

### Characterization of TGFβ3-preconditioned BMSC-EVs for chondrogenesis in vitro

BMSCs were isolated and identified as previously reported. Flow cytometry showed that BMSCs were positive for markers CD29, CD90, and CD105, but negative for markers CD34 and CD45 (Supplementary Fig. 1). Following the guideline recommended by ISEV (International Society for Extracellular Vesicles)^20^, EVs collected from BMSC media with TGFβ3 treatment were subject to miRNA sequencing. EVs were collected and added to BMSC culture to analyze their potential in chondrogenesis and OA treatment with mRNA sequencing. Collected EVs were characterized in terms of morphology, size, and surface markers (Fig. 1B–D and Supplementary Table 1). The typical cup-like shape with double-layer membrane structures ranging from 50 to 200 nm of the obtained EVs was observed with cryo-TEM analysis (Fig. 1B, C). As shown in Fig. 1D, the un-TGFβ3-preconditioned EV group (un-EVs) and TGFβ3-preconditioned EV group (T3-EV) were positive for EV markers including CD9, CD63, CD81, and Alix with western blotting assay. (Fig. 1D, Supplementary Table 1 and Supplementary Fig. 8) Native BMSCs were incubated with T3-EV and un-EVs for 6 h, and the PKH26-labeled EVs were observed surrounding the nucleus of recipient cells, indicating native BMSCs could take in the EV components (Fig. 1E). After incubation with different EVs, recipient BMSCs were subjected to chondrogenic culture for 21 days. Histological examination demonstrated that T3-EV and TGFβ3 treatment yielded cartilaginous matrix that stained positive for safranin-O and toluidine blue, indicative of the formed proteoglycan-rich, cartilage-like ECM (Fig. 1F). The phenomenon posed stark contrast with the results of un-EV and control groups, where little red granule could be observed or the bluish violet granules were sparsely scatted, suggesting the lagged extent of chondrogenic differentiation within 21 days compared with T3-EV and TGFβ3 groups. As for the expression of chondrogenic proteins, including SOX9, ACAN, and COL2A1 in T3-EV and TGFβ3, it had significantly upregulated compared with the un-EV and control groups (Fig. 1G and Supplementary Fig. 2 for each channel). In addition, qRT-PCR was further conducted to detect the expression of chondrogenesis-related genes (SOX9, aggrecan, and collagen II), and it was found that relevant genes of T3-EV and TGFβ3 groups also upregulated, remarkably higher than those in the un-EV and control groups (Fig. 1H and Supplementary Table 2). Cartilaginous tissues generated in T3-EV and TGFβ3 groups deposited rich GAGs (Fig. 1I) and expressed abundant ACAN and type II collagen (Fig. 1J, K). These results suggest that T3-EV could serve as a biosafe substitute for TGFβ3 to promote chondrogenesis in vitro, indicating its potential in cartilage regeneration and OA therapy.

### Discovery of T3-EV-associated miRNAs

**Fig. 2:**
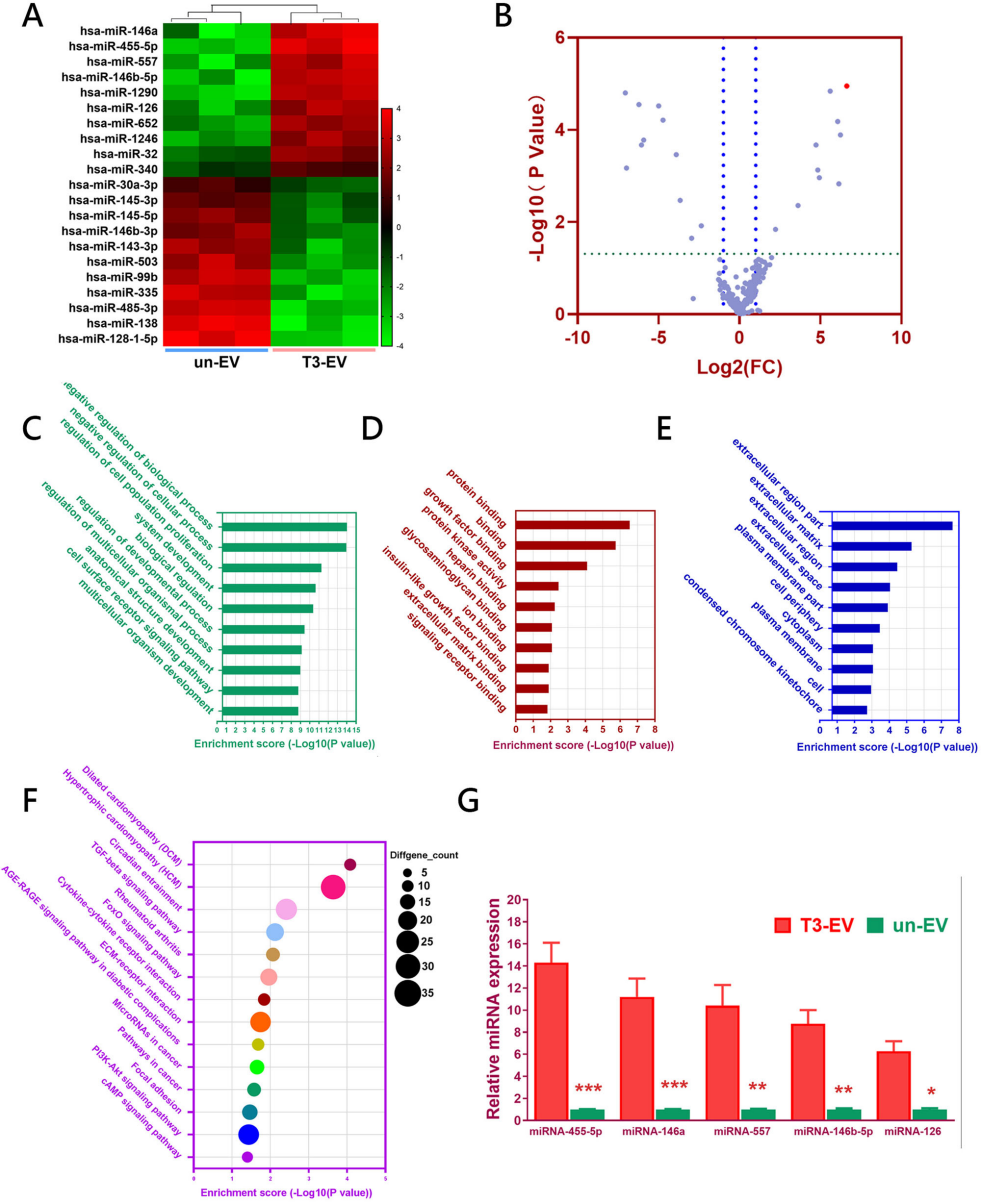
Discovery of T3-EV-associated miRNAs by microarray. **A** Heatmap of clustering dysregulated miRNA expression profiles with microarray in T3-EVs compared to untreated un-EV control. **B** Volcano plot of miRNA expression profiles and miR-455 (red dot) was most significantly upregulated in T3-EV. **C–E** All differentially expressed miRNAs(DEG with fold change >2 or <0.5, *p* value <0.01) were subjected to gene ontology (GO) analysis for **C** biological processes, **D** molecular function, and **E** cellular component. **F** Significantly enriched pathways for target genes of miRNAs enriched within T3-EV in Kyoto Encyclopedia of Genes and Genomes (KEGG) pathways. **G** miRNAs expression elevation (*n* = 3 for each) with the same EVs was validated with qRT-PCR. *P < 0.05, **P < 0.01, ***P **<** 0.001, NS not significant.

To further study the underlying mechanism of TGFβ3-sEV-mediated chondrogenesis, miRNA expression profiles of T3-EV and un-EV were analyzed and compared with high-throughput miRNA sequencing. The hierarchical cluster indicated that the EV-contained miRNA cargoes in the T3-EV group were obviously different from un-EV miRNA cargoes (Fig. 2A). In specific, 10 miRNAs, including hsa-miR-146a, hsa-miR-455-5p, hsa-miR-557, hsa-miR-146b-5p, and hsa-miR-126 were significantly upregulated in the T3-EV group, while 11 miRNAs, including hsa-miR-128-1-5p, hsa-miR-138, hsa-miR-485-3p, has-miR-335, and hsa-miR-99b, were downregulated compared with un-EV. Among the identified specific miRNAs in T3-EV, five of ten (50%) were previously reported to involve in chondrogenesis and OA development (including hsa-miR-146a, hsa-miR-455-5p, hsa-miR-146b-5p, hsa-miR-126 and hsa-miR-1246)^21–25^. Volcano plot of miRNA expression profiles demonstrated that miR-455 was significantly upregulated in T3-EV samples. Then Targetscan, miRanda and miRmap, PITA, and PicTar were used to predict the target genes. All differentially expressed miRNAs(DEG with fold change >2 or <0.5, *p* value <0.01) were subjected to gene ontology (GO) analysis for biological processes, molecular function, and cellular component. Predicted target genes of the differentially enriched miRNAs in T3-EV were involved in a broad range of biological functions, such as regulation of cell population proliferation, growth factor binding, glycosaminoglycan binding, and extracellular matrix binding (Fig. 2C–E). KEGG Pathway enrichment analysis based on the predicted target genes was also conducted, and the results were closely associated with the Circadian rhythm, TGF-beta signaling pathway, Rheumatoid arthritis, FOXO signaling pathway, Cytokine-cytokine receptor interaction, ECM-receptor interaction, MicroRNAs in cancer, focal adhesion, and PI3K-Akt signaling pathway (Fig. 2F). Then, the expression of the five most abundant miRNAs in T3-EV vs un-EV was further validated by qRT-PCR, and the results further confirmed T3-EV miRNA profiles in miRNA sequencing. (Fig. 2G).

### T3-EV enhanced chondrogenesis by modulating FOXO signaling pathway

**Fig. 3:**
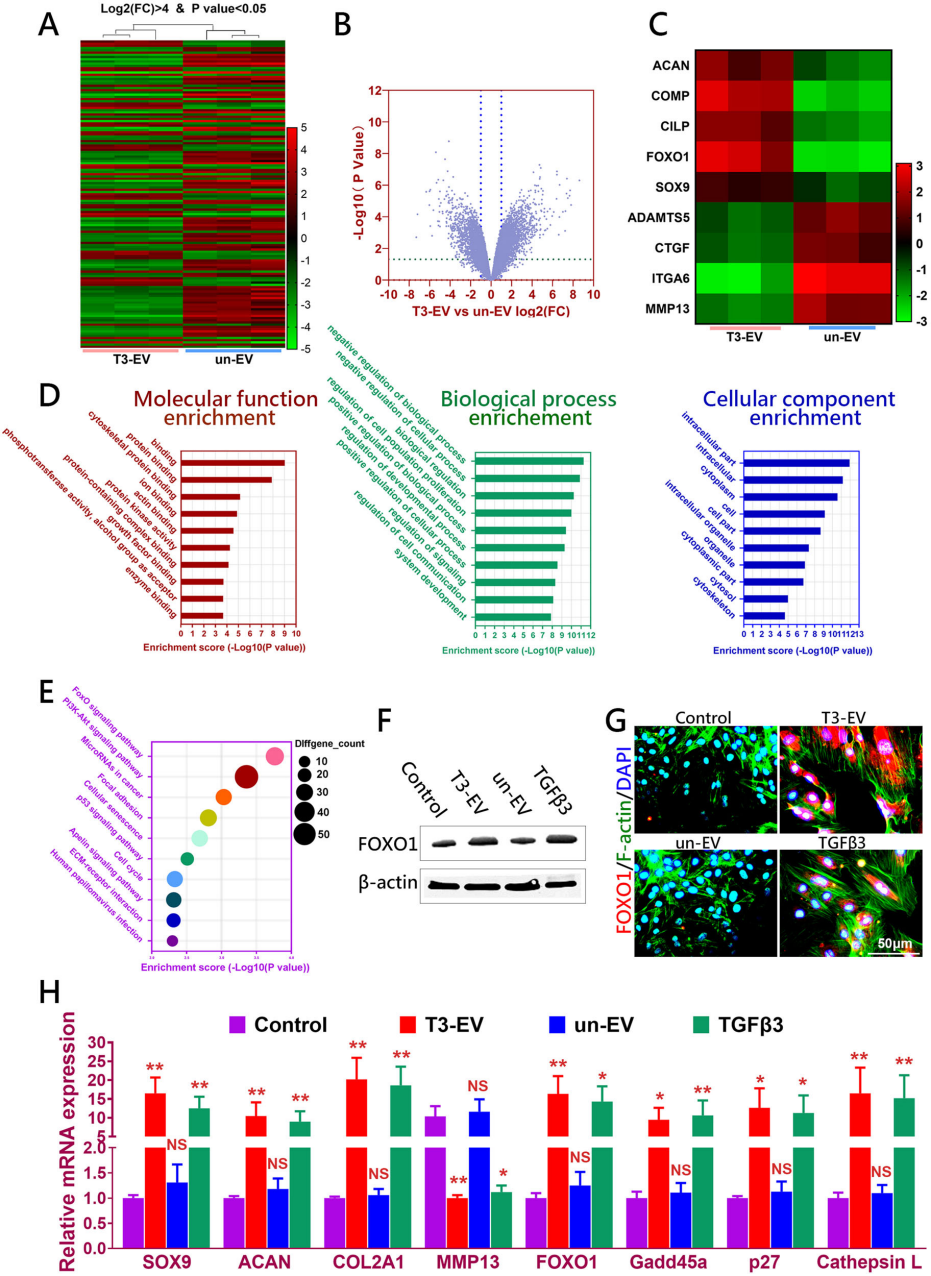
Activation of FOXO signaling by T3-EV. **A** Heatmap of clustering dysregulated mRNA expression profiles with microarray in T3-EV-treated BMSCs compared to un-EV treated control. **B** Volcano plot of mRNA expression profiles in T3-EV-treated BMSC recipient. **C** Dysregulated typical chondrogenic markers derived from the microarray results with T3-EV treatment. **D** All differentially expressed genes were subjected to gene ontology (GO) analysis (DEG with fold change >2 or <0.5, *p* value <0.01). BP biological processes, MF molecular function, CC cellular component. **E** Significantly enriched pathways for dysregulated genes enriched with T3-EV treatment in KEGG pathways. **F**, **G** FOXO1 expression(red) assayed with western blotting and fluorescent immunostaining (red for FOXO1; green for cytoskeleton) in T3-EV treated BMSC recipient. Cells were counterstained with DAPI for the nucleus (blue). Treatment with saline served as control. **H** Quantification of gene expression with the same BMSCs and the same EVs (*n* = 3 for each) with qRT-PCR for Chondrogenic genes (SOX9, ACAN, COL2A1, and MMP13) and FOXO signaling-related genes (FOXO1, Gadd45a, p27, and Cathepsin L). *P < 0.05, **P < 0.01, ***P < 0.001, NS not significant.

To test the chondrogenic potential of T3-EV on recipient BMSCs, mRNA profiles of recipient BMSCs with different EV treatment were comparatively analyzed with mRNA sequencing. Differentially regulated mRNAs were identified in the T3-EV group (Fig. 3A, B). Chondrogenesis-related genes (ACAN, COMP, CILP, FOXO1, and SOX9) were significantly upregulated, while expression of genes related to OA and cartilage catabolism (ADAMTS5, CTGF, ITGA6, and MMP13) decreased in BMSCs with T3-EV treatment (Fig. 3C). All differentially expressed genes were subjected to gene ontology (GO) analysis (Fig. 3D). GO terms for biological processes, molecular function, and cellular component were related to growth factor binding, enzyme binding, biological regulation, regulation of cell population proliferation and regulation of cell communication (Fig. 3D). KEGG Pathway analysis based on the differentially expressed genes was conducted and showed closely associated with FOXO signaling pathway, PI3K-Akt signaling pathway, MicroRNAs in cancer, focal adhesion, Cellular senescence, p53 signaling pathway, cell cycle, and ECM-receptor interaction (Fig. 3E). FOXO signaling pathway was also enriched in the results of KEGG analysis with predicted target genes. Previous studies have indicated the significance of the FOXO signaling pathway in OA development and chondrogenesis. Hence, we chose to evaluate FOXO1 expression in T3-EV mediated chondrogenesis with immunoblotting and immunofluorescence. FOXO1 expression significantly increased with T3-EV treatment in BMSCs compared to the control group and the un-EV group. (Fig. 3F–H and Supplementary Figs. 3, 4, 8). T3-EV decreased the adipogenic potential of BMSCs compared to the control group. (Supplementary Fig. 4) Compared to the enhanced osteogenic marker expression by un-EV and TGFβ3, T3-EV maintained the low expression of osteogenic genes, indicating a decrease in the osteogenic commitment of BMSC by T3-EV treatment. Meanwhile, T3-EV had minimal effects on pluripotent markers’ expression, which were significantly decreased with TGFβ3 treatment in comparison. Meanwhile, T3-EV not only enhanced chondrogenesis by elevating the expression of chondrogenesis-related genes, but also significantly boosted the expression of downstream genes in the FOXO signaling pathway (Fig. 3H). These results indicated that T3-EV exerted chondrogenic effects by activating FOXO signaling pathway, and FOXO1 might be a potential downstream target for the enriched miRNAs in T3-EV.

### SOX11 was identified as a target gene for miR-455

**Fig. 4:**
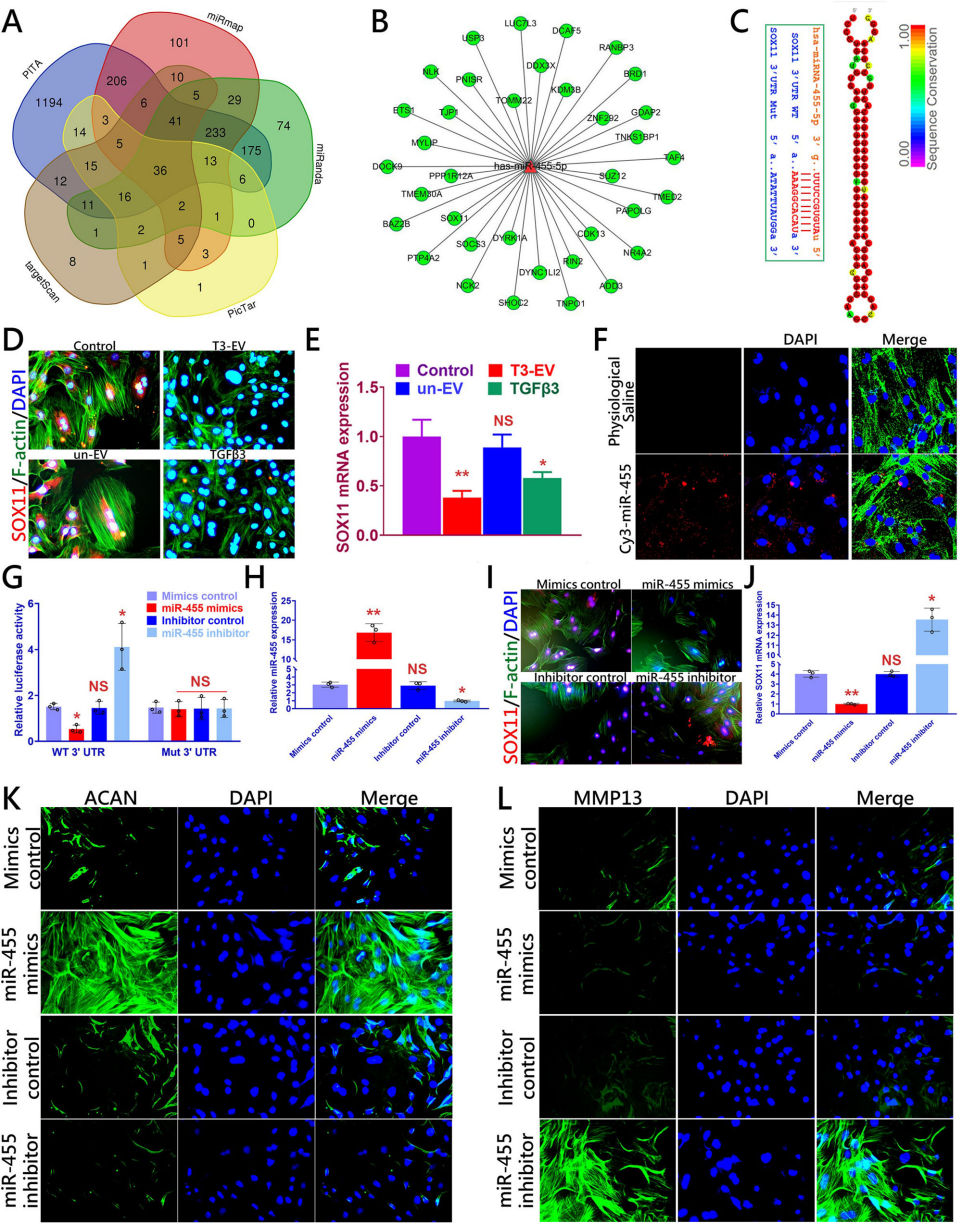
SOX11 is a direct target of miR-455 to modulate chondrogenesis. **A** All predicted genes were compiled for Venn analysis to search for the potential targets of miR-455. **B** miRNA–mRNA network using the Cytoscape software was constructed for miRNA-455. **C** Sequence of wild-type (WT) and mutant (Mut) SOX11 binding sites for miRNA-455 (left) and conservation level of miR-455 sequence among species (right). **D**, **E** SOX11 expression(red) assayed with fluorescent immunostaining (red for SOX11; green for cytoskeleton) and qRT-PCR in T3-EV treated BMSC recipient (*n* = 3 for each with the same BMSCs and the same EVs). **F** Fluorescence micrograph of Cy3 (red)-labeled miR-455 mimic internalized by BMSCs. **G** Luciferase reporter assay analysis results (*n* = 3 for each) to confirm the direct interaction between miR-455 and SOX11 binding sites. Relative luciferase reporter activity was assessed for co-transfected SOX11 WT (or Mut) with miR-455 mimics or inhibitors in cultured primary BMSC cells. miRNA-455 mimics control and inhibitor control served as negative controls. **H–J** miR-455 and SOX11 expression(red) with **H**, **J**) qRT-PCR and **I** fluorescent immunostaining in BMSCs transfected with miR-455 mimics or inhibitor (*n* = 3 for each). **K**, **L** Fluorescent immunostaining was also conducted on anabolic and catabolic markers ACAN (green) and MMP13 (green) in BMSCs transfected with miR-455 mimics or inhibitors. Cells were counterstained with DAPI for the nucleus (blue). *P < 0.05, **P < 0.01, ***P < 0.001, NS not significant.

MiR-455 has been reported to participate in chondrogenesis and OA development in previous studies. To search for the potential target genes of miR-455, we collected all the predicted potential target genes for Venn analysis (Fig. 4A). Moreover, a miRNA–mRNA network using the Cytoscape software was constructed (Fig. 4B). Based on these results, SOX11, a negative regulator of SOX9 expression and chondrogenesis, was predicted as a target gene of miR-455 (Fig. 4B, C). Additionally, miR-455 shows a high level of sequence conservation among different species (Fig. 4C). Immunostaining and qPCR also demonstrated significantly depressed SOX11 expression in recipient BMSCs after being treated with TGFβ3 or T3-EV (Fig. 4D, E). To further confirm the functional interaction between miR-455 and SOX11, we performed a luciferase reporter assay analysis (Fig. 4F–G). Fluorescence demonstrated internalization of Cy3 (red)-labeled miR-455 mimic taken by BMSCs (Fig. 4F). Co-transfected SOX11 WT (WT) with miR-455 mimics in cultured primary human BMSCs showed that relative luciferase reporter activities were significantly lowered when compared with cells transfected SOX11-mut (mutant) with miR-455 mimics (Fig. 4G). Upregulation of miR-455 by miR-455 mimics significantly reduced SOX11 expression while miR-455 inhibitor significantly increased SOX11 expression in cultured BMSCs. (Fig. 4H–J). Next, we further analyzed the chondrogenic effect of miR-455 expression on anabolic/catabolic markers. The expression of Aggrecan (ACAN) was increased in cultured BMSCs that were transfected with miR-455 mimics. In contrast, overexpression of miR-455 strongly decreased MMP13 levels, whereas inhibition of miR-455 increased MMP13 levels in BMSCs (Fig. 4K, L). Collectively, the data validated SOX11 as a direct target of miR-455, and upregulation of miR-455 promotes the chondrogenesis of BMSCs.

### MiR-455 regulates chondrogenesis and OA development by modulating SOX11/FOXO signaling pathway

**Fig. 5:**
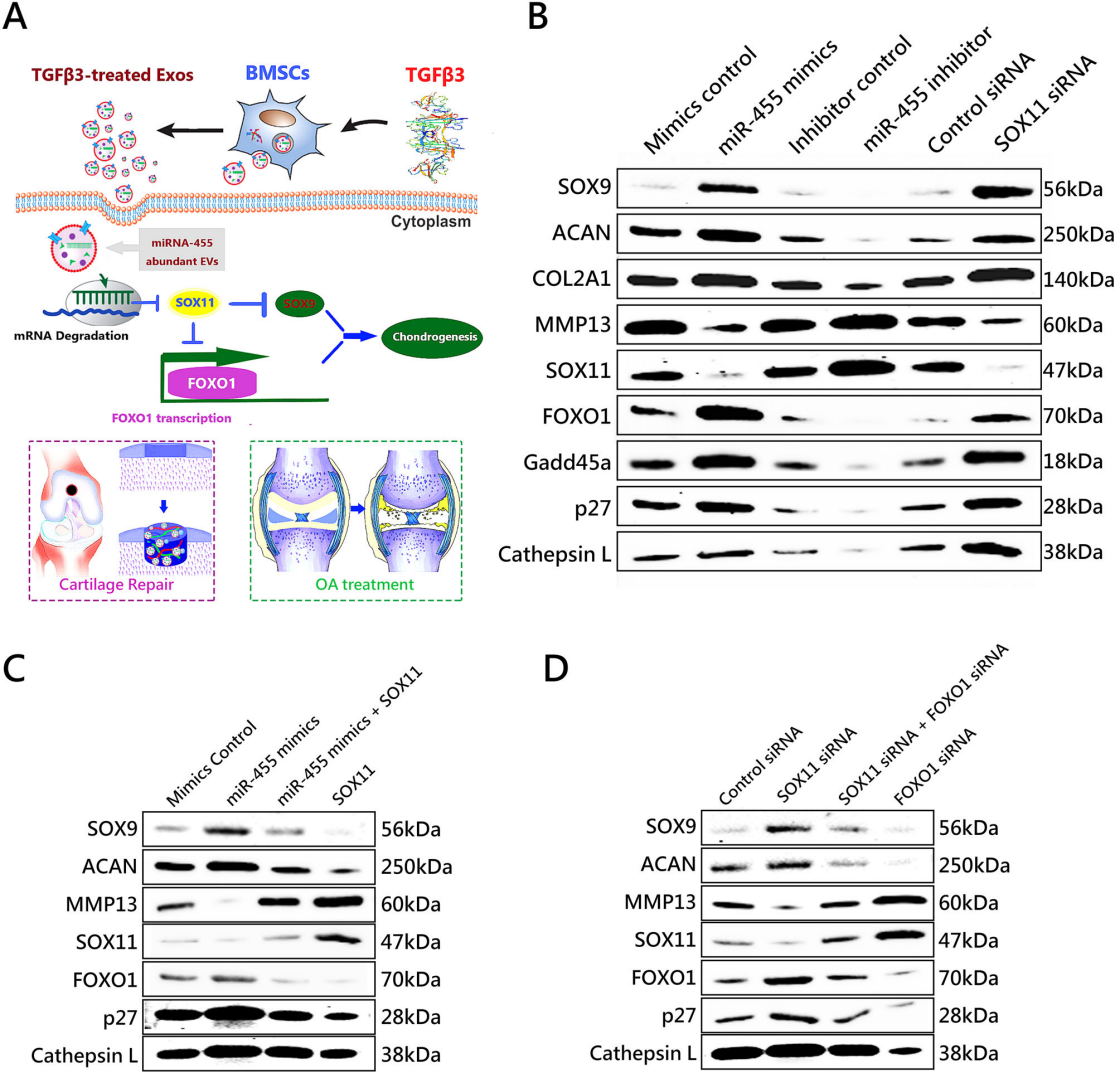
MiR-455 regulates chondrogenesis and OA development by modulating the SOX11/FOXO signaling pathway. **A** Schematic representation of how the miR-455/SOX11/FOXO signaling pathway might mediate chondrogenesis and the therapeutic effects of T3-EV in OA treatment and cartilage regeneration. **B** Cultured primary human BMSCs were transfected with miR-455 mimics, miR-455 inhibitor, their negative controls, control siRNA or SOX11 siRNA for 72 h, respectively, and the expression levels of chondrogenic markers SOX9, ACAN, COL2A1, MMP13, and SOX11/FOXO signaling pathway markers SOX11, FOXO1, Gadd45a, p27, and Cathepsin L were assessed with western blot. **C** Rescue experiments was established in cultured primary BMSCs to validate the relationship between miR-455 and SOX11. Elevation of SOX9 and ACAN expression levels by miR-455 mimics was rescued by restoration of SOX11 expression. In comparison, inhibition of SOX9, ACAN, and FOXO1 expression by SOX11 overexpression was rescued by miR-455 mimics. **D** Upregulation of SOX9 and ACAN expression levels by SOX11 siRNA was abolished by silencing of FOXO1 expression. In comparison, upregulation of FOXO1 target genes (p27 and Cathepsin L) expression by FOXO1 siRNA was abolished by silencing of SOX11 expression.

Further experiments were conducted to explore the mechanism of chondrogenesis by T3-EV enriched miR-455/SOX11 signaling axis (Fig. 5A). As shown in Fig. 5B, the FOXO signaling pathway was significantly enriched for T3-EV in KEGG pathways (Fig. 3E). FOXO signaling pathway has been identified in chondrogenesis and joint degeneration^26^. The findings that miR-455 promotes chondrogenesis by SOX11 prompted us to investigate the potential association between miR-455 and SOX11/FOXO signaling pathway. (Fig. 5, B–D and Supplementary Figs. 5, 8) Cultured primary human BMSCs were transfected with miR-455 mimics, miR-455 inhibitor, or their negative control, respectively. Expression levels of MMP13 and SOX11 were significantly decreased in BMSCs that stably overexpress miR-455 (Fig. 5B and Supplementary Figs. 5, 8). In contrast, expression of MMP13 and SOX11 were upregulated in BMSCs transfected with miR-455 inhibitor (Fig. 5B–D and Supplementary Figs. 5,8). Moreover, miR-455 mimics activated FOXO1 expression and its downstream target genes. Besides, SOX11 small interfering RNA (siRNA) had effects on ACAN, SOX9, MMP13, SOX11, FOXO1, Gadd45a, p27, and Cathepsin L genes similar to the effects induced by miR-455 (Fig. 5B), indicating that miR-455 regulates chondrogenesis and OA progression by targeting the SOX11/FOXO signaling pathway. Further experiments were performed to validate the relationship between miR-455 and SOX11/FOXO signaling. (Fig. 5C, D). These results indicate that miR-455-mediated chondrogenesis is primarily through the SOX11/FOXO axis. MiR-455 overexpression downregulated SOX11 expression, which further enhanced FOXO1 transcription and upregulated SOX9 expression (Fig. 5C). Meanwhile, blocking FOXO1 could partially attenuate the activation of FOXO signaling by downregulating SOX11, consequently inhibiting SOX9 transcription as well as chondrogenesis (Fig. 5D).

### Cartilage defect repaired by T3-EV BMSC hydrogel in vivo

**Fig. 6:**
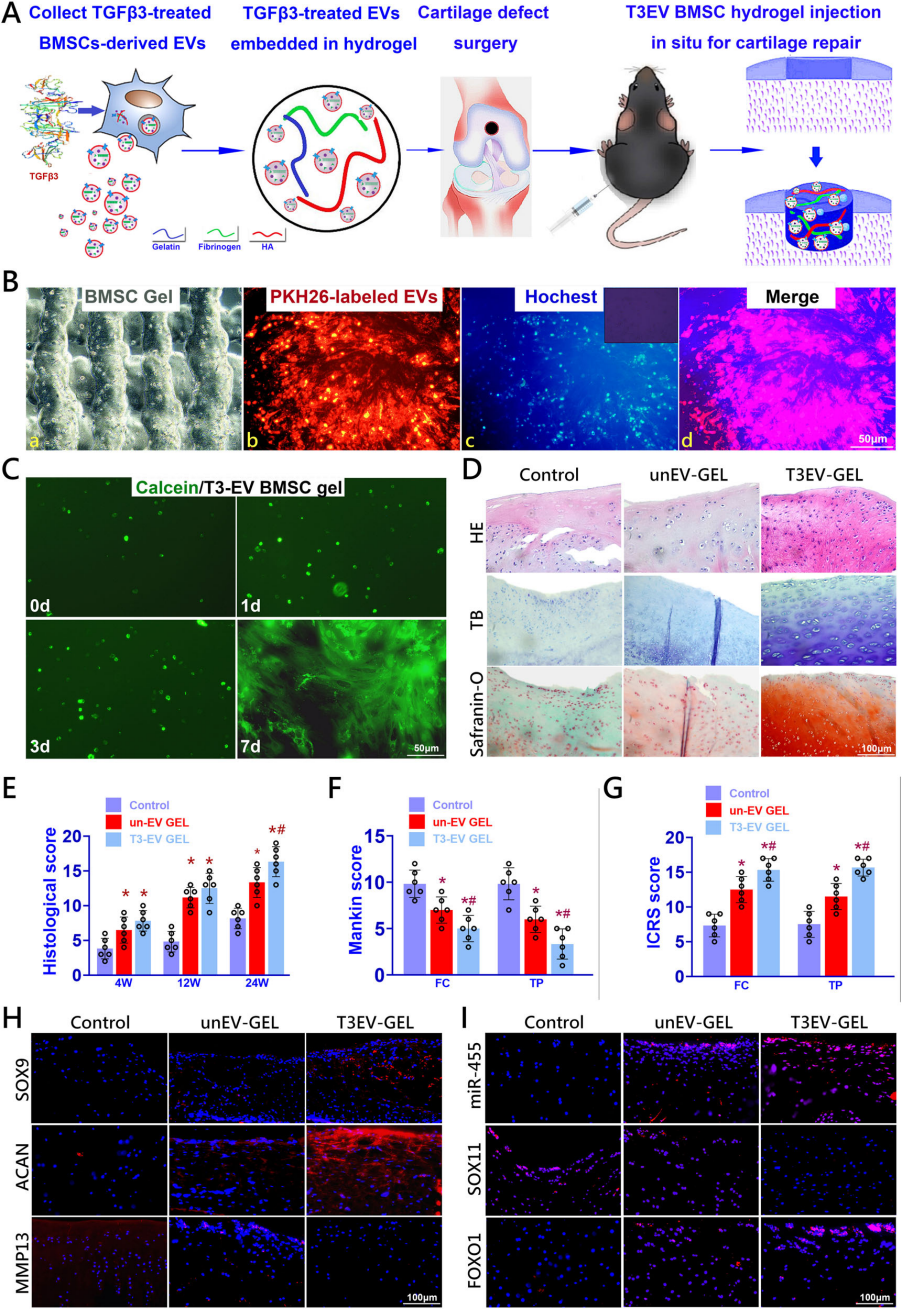
Injection of T3-EV-conjugated composite hydrogel for cartilage regeneration in vivo. **A** Rat T3-EV were derived for T3-EV hydrogel formation and delivery. T3-EVs were derived for embedding into composite BMSC hydrogel produced with a mixture of gelatin, fibrinogen, and HA. Cartilage defect injury was created, and hydrogel injection was performed in situ to deliver T3-EV hydrogel for defect repair. **B** Before transplantation, T3-EV hydrogel was cross-linked by the addition of thrombin to further maintain the shape fidelity of the hydrogel. (a) The cross-linked hydrogel demonstrated good shape fidelity and b–d) good distribution of EVs internalized within BMSCs (b: PKH26-labeled EVs stained red; c: nucleus stained blue; d: merged image with b and c). **C** Cell viability >95% was demonstrated for 7 days with live/dead assay for the T3-EV hydrogel. **D** Histological assessment of cartilage repair with T3-EV hydrogel gel at 24 weeks. Neo-cartilage formation in different groups was compared with tissue integrity (HE staining in the 1st row) and GAG deposition with Toluidine blue and Safranin-O staining (second and third row). **E–G** Histological grading of repaired cartilage in different groups (*n* = 6 for each) over 24 weeks. **F** Articular cartilage in both un-EV-gel and T3-EV-gel groups showed declined Mankin score and **G** higher ICRS histological score compared to the control group in the femoral condyle (FC) and tibial plateau (TP) over the 24 weeks in vivo. **H** Immunostaining of chondrogenic markers SOX9 (first row), ACAN (second row), and MMP13 (third row) were also conducted and observed (red for the protein, blue for the nucleus) in the generated cartilage in different groups. **I** Immunostaining of miR-455 (FISH), SOX11, and FOXO1 was also performed and observed (red for the protein, blue for the nucleus) to examine the miR-455/SOX11/FOXO axis in the generated cartilage in different groups. *P < 0.05, **P < 0.01, ***P < 0.001, NS not significant. #*p* < 0.05 compared to the un-EV gel group.

T3-EVs were collected from TGFβ3-treated BMSCs for later embedding and composite hydrogel conjugated with T3-EVs and BMSCs was constructed for cartilage repair in rat knee joints (Fig. 6A and Supplementary Table 3). Rat BMSCs were derived for cell delivery in the hydrogel as previously reported^7^. Before transplanting into the defect site, T3-EV BMSC hydrogel was cross-linked by thrombin addition to maintain its shape fidelity. (Fig. 6B, a). The release rate of EV from the hydrogel bioink was measured for a month (Supplementary Fig. 6). PKH26-labeled EVs were successfully embedded in the hydrogel and showed well-proportioned cellular uptake with live/dead assay (Fig. 6B, b–d). Live/dead assay demonstrated >95% cell viability and nice cell spreading at day 7 in the hydrogel (Fig. 6C). As previously reported, full-thickness cartilage defect was created in the rat knee (Supplementary Fig. 7). Cartilage repair with TGFβ3-treated BMSC-derived EV-conjugated hydrogel (TBEH) showed significantly better cartilage regeneration at 24 weeks compared to the control and untreated-BMSC-derived EV-conjugated hydrogel (UBEH) group (Fig. 6D, first row). Neo-cartilage in the TBEH group presented with greater GAG deposition and better tissue integrity of both the repaired cartilage and the cartilage-bone interface. (Fig. 6D, 2nd–3rd rows and Supplementary Fig. 7). Articular cartilage in both control and un-EV gel groups showed declined ICRS score and higher Mankin histological score compared to the T3-EV gel group, indicating better chondroprotective effects with T3-EV gel over 24 weeks in vivo (Fig. 6E–G). In comparison with the control group, stronger SOX9 and ACAN immunostaining were observed in newly-generated cartilage in the T3-EV gel group, demonstrating successful reconstruction of articular cartilage with abundant ECM deposition (Fig. 6H). Moreover, MMP13 expression was significantly decreased by T3-EV gel transplantation, restoring the balance in focal chondrocyte anabolism and catabolism (Fig. 6H). In evaluating the modulation of the miR-455/SOX11/FOXO signaling axis during cartilage repair induced by T3-EV gel transplantation, upregulation of miR-455 and FOXO1 was observed with immunostaining in the neo-cartilage tissue from the T3-EV gel group. Furthermore, T3-EV gel transplantation effectively inhibited SOX11 expression in the repaired cartilage (Fig. 6I). Taken together, these results provided promising evidence that T3-EV gel transplantation not only showed better cartilage repair but better maintenance of joint function by modulating the miR-455/SOX11/FOXO signaling axis.

### Intra-articular T3-EV injection reversed OA development by regulating the miR-455/SOX11/FOXO signaling axis

**Fig. 7:**
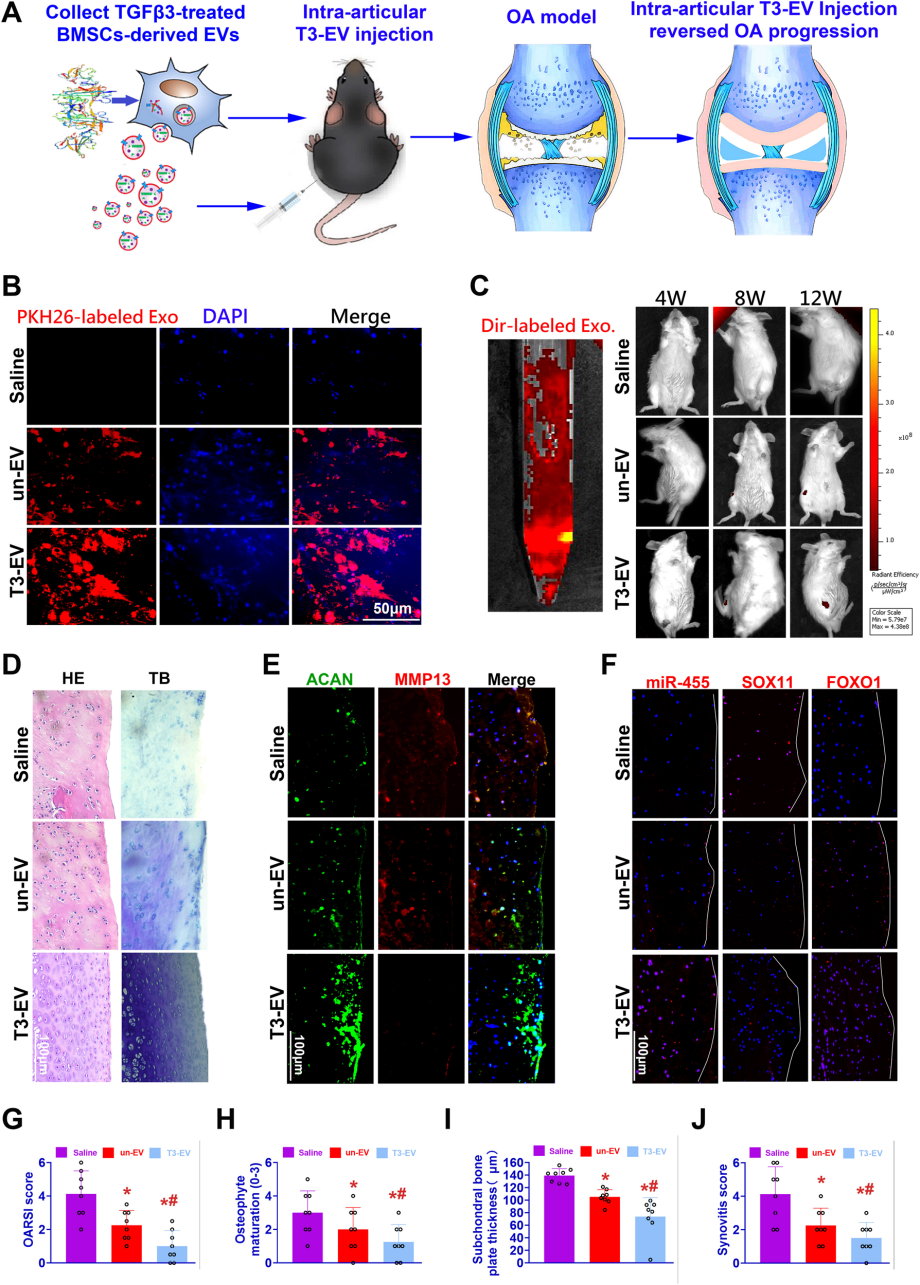
Intra-articular T3-EV injection reversed OA progression by regulating the miR-455/SOX11/FOXO signaling axis. **A** To determine whether T3-EV transplantation could reduce or reverse the progression of OA, intra-articular injection of T3-EV labeled with Dir was performed for rats with DMM surgery. **B** PKH26-labeled EVs (red) to detect their internalization by BMSC in vitro. **C** Intra-articular delivery of the Dir-label T3-EVs (left: red) were monitored with in vivo fluorescence imaging to evaluate the near-infrared imaging effect and distribution of EVs for 12 weeks, showing an ideal intra-articular delivery effect (right). **D**–**F** Histological assessments of joint cartilage with **D** HE (left column), TB staining (right column), and **E**, **F** immunostaining for ACAN, MMP13, miR-455, SOX11, and FOXO1 in different groups. **G–J** Quantification and comparison of histological grade for OA progression in different groups (*n* = 8 for each). Joint destruction severity was determined with OARSI score, osteophyte formation, subchondral bone plate thickness, and synovial inflammation as previously reported^60–62^. Saline: OA model group with saline injection; un-EV: only untreated EVs were injected for OA treatment; T3-EV: TGFβ3-treated EVs were injected for OA treatment. *P < 0.05, **P < 0.01, ***P < 0.001, NS not significant. #*p* < 0.05 compared to the un-EV gel group.

To determine whether T3-EV would reduce or reverse the OA progression, intra-articular injection of Dir-labeled T3-EV was performed for rats with ACLT surgery (Fig. 7A). Cellular uptake of PKH26-labeled EVs was demonstrated in vitro compared to the saline group (Fig. 7B). EVs were administered with intra-articular injection for six consecutive weeks from the 5th week after ACLT surgery. Intra-articular delivery of Dir-labeled EVs were monitored in vivo in real-time protein and showed an ideal intra-articular delivery effect (Fig. 7C). Compared to the control group presented with severe cartilage erosion, osteophyte formation and synovitis, local delivery of T3-EV remarkably protected the structural integrity of articular cartilage, proven by histological assessments and proteoglycan depositions (Fig. 7D–J). Moreover, MMP13 expression was significantly decreased by T3-EV transplantation, while abundant ACAN expression was noted (Fig. 7E). MiR-455 and FOXO1 expression was significantly downregulated with immunostaining in the OA cartilage tissue from the control group. To compare, T3-EV injection significantly increased miR-455 and FOXO1 expression in the joint cartilage (Fig. 7F). Furthermore, in contrast to the increased expression level of SOX11, the target gene of miR-455 in OA cartilage, T3-EV treatment effectively declined SOX11 expression (Fig. 7F). Taken together, these results provided promising evidence that intra-articular injection of T3-EV might be an effective therapeutic option for OA prevention, highlighting the importance of miR-455/SOX11/FOXO signaling axis as a potential therapeutic target in OA treatment.

## Discussion

In our present study, we have demonstrated EVs derived from TGFβ3-preconditioned BMSCs could promote chondrogenesis and alleviate OA progression by upregulating FOXO signaling pathway with the T3-EV enriched miRNA-455/SOX11 axis. Our findings expand the understanding of chondrogenesis and OA development modulated by EV-miRNAs, and provide novel means to feasibly apply engineered EVs from chondrogenic preconditioned-BMSCs for cartilage repair and OA treatment.

The potential applications of BMSCs for cartilage regeneration and OA treatment have been extensively studied before^4–9^. However, the single usage of BMSCs was far from satisfactory due to their low chondrogenic efficiency in vivo and the oncogenicity potential by their chondrogenic cocktails^27–29^. Our previous research indicated that TGFβ3-priming of BMSCs could generate hyaline cartilage in the articular surface^12,30^, and our findings in the present study further demonstrated that EVs derived from TGFβ3-primed-BMSCs could serve as an alternative for TGFβ3 and effectively replicate the natural chondrogenic process in vivo.

Therapeutic efficacy of EVs on cartilage repair has been previously reported in several studies^31,32^. It has shown that EVs could accelerate the process of neo-cartilage formation as well as enhance ECM synthesis and deposition. Specifically, by protecting chondrocytes from apoptosis and balancing the synthesis-degradation equilibrium within, BMSC-derived EVs have revealed the merits of anti-inflammation and re-establishing the homeostasis of cartilage^4,6,17,33–36^. However, limited cellular sources in the joint could also render the failure of single EV therapy in vivo, which need to be overcome in order to achieve cartilage regeneration. According to our previous study, we have demonstrated that high cellular density is essential to guaranteeing the successful regeneration of native joint cartilage^37^. Herein, in the present study, BMSC-laden T3-EV hydrogel ensures the high-densified and well-proportioned distribution of BMSCs and derived T3-EVs, and thus protects focal BMSC viability and promotes its differentiation and expansion in the hydrogel^7^. T3-EV BMSC hydrogel offers a stable microenvironment for the sustained release of embedded T3-EVs and chondrogenic differentiation of BMSCs within to form their secreted cartilage matrix that replaces the hydrogel as it slowly degrades^5^. However, the release of embedded EVs from the T3-EV BMSC hydrogel was not tracked in vivo after transplantation. Generally speaking, the intra-articular environment in vivo would definitely lead to faster hydrogel disintegration and EV release. In this case, the hydrogel scaffolding would offer a much more stable microenvironment to optimize intra-articular EV applications for OA treatment and cartilage regeneration.

EV-contained miRNA was a key cargo component of EV content to mediate its therapeutic potential by targeting downstream genes and signaling pathways^38,39^. Previous studies have suggested EV-miRNAs could contribute to joint protection and inflammation relieving by modulating cartilage and synovial functions^35,36,40–42^, indicating EV-transferred miRNA as a potential candidate in OA treatment and cartilage regeneration^43^. Synovium could be flamed in OA due to the long-term inflammation caused by cartilage injury, We believe the effects of EVs on synovium could be partially attributed to the reparative effects of EVs on the joint cartilage, which led to a subsequent reduction of synovial inflammation. In T3-EV, miR-455 was the most highly enriched one among several miRNAs, suggesting T3-EV might exert chondrogenic function by delivering miR-455^44–47^. Moreover, we showed that T3-EVs enriched with miR-455 were successfully taken up by the BMSC recipients, leading to its chondrogenic differentiation. Our study further identified SOX11 as the direct target gene for miR-455. In addition, miR-455 mimic suppressed the production of SOX11, whereas the mutant miR-455 upregulated SOX11 expression. SOX11 was a negative regulator in SOX9 transcription and SOX11 was reported to inhibit SOX9-mediated chondrogenesis^48–51^. In this study, we revealed that miR-455 regulated chondrogenesis and OA development by modulating the SOX11/FOXO signaling pathway^52–54^. T3-EV delivered miR-455 bound the 3ʹ-UTR of SOX11 and inhibited the expression of SOX11. Inhibitory SOX11 signal further led to upregulation of FOXO1 and activation of FOXO signal pathway, enhancing chondrogenesis of BMSC and extracellular matrix deposition. Taken together, T3-EV enriched miR-455/SOX11/FOXO signaling may offer a novel therapeutic target for OA treatment and cartilage regeneration based on this study.

In summary, we found that EVs derived from TGFβ3-preconditioned BMSCs rich in miR-455 promoted OA alleviation and cartilage regeneration by activating the SOX11/FOXO signaling pathway. Moreover, the designed T3-EV hydrogel showed great potential in cartilage defect treatment.

## Methods

### Cell culture and EV collection

Human BMSCs were isolated from biopsies of healthy clinical donors. BMSCs were isolated from bone marrow aspirates. Briefly, marrow aspirates (20 mL volume) were harvested and immediately transferred into plastic tubes. BMSC purity was characterized by flow cytometry. In brief, BMSCs were trypsinized and washed with phosphate-buffered saline (PBS) twice. The cell concentration was adjusted to 0.5 × 105 per tube. Cells were incubated with FITC-labeled antibodies against CD29, CD34, CD45, CD90, and CD105 (ab150002, ab78165, ab27287, ab124527, and ab11415, Abcam dilution at 1:2000) at 4 °C in the dark for 30 min. Cells were washed with PBS twice and centrifuged. The cells were fixed with 4% paraformaldehyde and analyzed in a FACS–Canto™ II Flow Cytometer (BD Biosciences). The BMSC pools were expanded until passage 3 for further treatment at 37 °C, 5% CO_2_, and 95% humidity in Dulbecco’s modified Eagle medium (DMEM, Life Technologies, China) with 10% fetal bovine serum (HyClone FBS, Thermo Scientific, USA), 1% antibiotic–antimycotic (100 units mL^−1^ penicillin, 100 mg mL^−1^ streptomycin, and 0.25 mg mL^−1^ amphotericin B), and 1 × 10^−3^ M sodium pyruvate (Life Technologies, China). The FBS had been centrifuged at 100,000 × *g* to eliminate preexisting bovine-derived exosomes. The Medium was changed every 2–3 days, and BMSCs were harvested with Trypsin (Life Technologies, China) at a confluence of 80–90%. TGFβ3(50 ng/ml) was added to the medium for the T3-EV group for 7 days before EV collection. Our previous findings have shown that 7 days of TGFβ3 preconditioning (50 ng/ml) can commit BMSCs to a pre-cartilaginous stage. For TGFβ3 preconditioning, ~2 × 10^6^ BMSCs were seeded into culture dishes for 12 h to achieve 70–80% confluence. Then, cells were incubated with a fresh growth medium containing 50 ng/ml of TGFβ3 for 7 days or a growth medium alone as a negative control. The Final concentration of TGFβ3 in the medium was kept at 40–70 ng/ml and the Medium was changed every 2 days. After preconditioning was completed, the medium was aspirated, and the cells were rinsed repeatedly to eliminate residual TGFβ3. The ethical committee for shanghai ninth hospital (school of medicine, Shanghai Jiao Tong University) approved all procedures, and patients’ informed consent forms were obtained. EVs were then isolated from this conditioned medium (CM) by a multi-filtration system based on the tangential flow filtration system (TFF). Collected CM (500 mL) was centrifuged at 300×*g* for 10 min to remove cell debris. The resulting supernatant was filtered using a 0.4-μm cell strainer and 0.22-μm bottle top filter to eliminate micro-vesicles. To remove soluble proteins and antibiotics, the suspension was subjected to TFF with a 300 kDa MWCO capsule. The suspension was continuously circulated through the membrane filter system and concentrated at 4 mL/min of operation speed. Subsequently, PBS was added to the suspension, and the cycle of TFF was repeated to remove residual soluble proteins in the concentrated EV solution. EVs were obtained in a final volume of ~10 mL. The EVs were stored in −70 °C freezers until use. The EVs released profiles from hydrogels were measured using a BCA protein assay kit (Beyotime, China). Briefly, the hydrogels were immersed in PBS in a 24-well plate. At specific time points, the surface supernatant was collected and fresh PBS was added. The released EVs were quantified and expressed as the release percentage. Data were presented as mean ± SD of three replicates (Supplementary Fig. 3).

### Transmission electron microscopy

To verify the presence of intact EVs, transmission electron microscopy image analysis was performed. EVs were fixed with 0.5% glutaraldehyde solution overnight. The fixed EVs were centrifuged at 13,000 × *g* for 3 min. Then the supernatant was removed. Next, the samples were dehydrated in absolute ethanol for 10 min and placed on formvar-carbon-coated copper grids (TED PELLA, Inc., Redding, CA, USA). The grids were contrasted with 1% phosphotungstic acid for 1 min and then washed several times with absolute ethanol solution. The grids were dried off completely and then examined with a JEM-2100 F field emission electron microscope (JEOL Ltd., Japan). For cryo-TEM of the EVs, the EVs were added onto the lacey carbon grid (Electron Microscopy Science, Hatfield, PA, USA). The grid was frozen in liquid nitrogen. The samples were analyzed with a Tecnai F20 Twin transmission electron microscope.

### Dynamic light scattering (DLS) and nanoparticle tracking analysis (NTA)

The sizes of EVs were measured by DLS performed with Zetasizer Nano ZS90 (Malvern, Worcestershire, UK). EVs resuspended in PBS were placed in a UV-transparent cuvette (Sarstedt AG & Co., Germany). The performed analyses were repeated at least three times, and the mean values were reported. Immediately after the isolation of EVs, the particle concentration was measured with Nanosight LM10 (Malvern Instruments Ltd., Malvern, UK). Samples were diluted in PBS to obtain a concentration within the recommended measurement range (20–30 particles/frame), corresponding to dilutions from 1:10 to 1:100 depending on the initial sample concentration. The software settings for analysis were as follows: detection threshold 3; temperature between 20 and 23 °C; the number of frames 30 and measurement time 30 s. The size distribution and particle concentration each represent the mean of three individual measurements.

### EV treatment of human BMSC

Human BMSCs were seeded onto a 24-well culture plate at 4 × 10^4^ cells per well and cultured for 24 h at 37 °C under 5% CO_2_. The cells were then washed with PBS and treated with T3-EV or untreated-EV (1 × 10^8^, 2 × 10^8^ particles/mL) for 7 days. Saline was used in the control group and TGFβ3 (50 ng/ml) was used in the TGFβ3 group. EV or TGFβ3 was added at each media change every 2–3 days. Cells were treated for 7 days for immunofluorescence and for 21 days for all the other assays. Cells were then stained with toluidine blue and safranin-O according to standard protocols. Immunofluorescent staining of chondrocyte markers SOX9, ACAN, and COL2A1 (ab185966, ab232628, and ab34712, Abcam, dilution at 1:1000–2000) were conducted according to standard protocols in the generated cartilage tissues in different groups. The stained images were taken using a light microscope. Differentially expressed miRNAs were further validated by quantitative reverse transcriptase–polymerase chain reaction (qRT-PCR). GAGs and types I and II collagen were quantitatively assayed (6 vs 6) and normalized to DNA content. The samples were washed with PBS and digested by papain solution (1 mg/mL papain, 10 mM Na2EDTA, and 10 mM l-cysteine hydrochloride dissolved in 0.1 M PBS) for 16 h at 57 °C. After digestion, the samples were centrifuged at 12,000 g for 3 min. The GAG content of each sample (*n* = 3) was quantitated by using a 1, 9-dimethyl methylene blue dye-binding assay (DMMB, Sigma), using a standard curve generated by chondroitin sulfate B. The absorbance was read at a wavelength of 525 nm. The DNA content in each sample lysate was determined by a PicoGreen® dsDNA quantitation assay. Collagen I and II were quantified according to the protocol with the Human collagen I and II ELISA Kit (Cat#: EKF57573, *Biomatik*; Cat#::ab210966, *Abcam*). The GAG and collagen content was then normalized to the DNA content. The assay was performed in triplicate.

### Cellular uptake of EVs

EVs were labeled with PKH26 (Sigma, St. Louis, MO, USA) according to the manufacturer’s protocol. To remove free dye aggregates, the PKH26-labeled EV suspension was purified with density gradient centrifugation. The centrifugation details were used according to PKH26 staining dye protocols. Briefly, EVs were incubated with 500 ul Dilution C solution and 4ul PKH26 dye solution for 5 min at room temperature in a dark environment. Then, 500 ul 1% bovine serum albumin was added to stop the process of staining. The labeled EVs were obtained after being centrifuged at 100,000 × *g* for 70 min, 400 × *g* for 10 min, and then 400 × *g* for 5 min and resuspended with 200 ul cold PBS. Recipient BMSCs were incubated with 5 × 10^8^ particles/mL of PKH26-labeled EVs for 12 h and then observed by confocal laser scanning microscopy (CLSM; Zeiss, Weimar, Germany)

### Microarray analysis

Total RNA was extracted from extracellular vesicles using the exoEasy Qiagen kit according to the manufacturer’s instructions. Microarray analysis of six cell samples (mRNA microarray for three T3-EV-treated BMSC and three un-EV-treated BMSC samples) and six EV samples (miRNA microarray for three T3-EV and three un-EV samples) was performed using an Agilent mRNA Microarray Kit, Release 21.0, 8 × 60K (Agilent Technologies, CA, USA). The study is compliant with the Guidance of the Ministry of Science and Technology (MOST) for the Review and Approval of Human Genetic Resources. Total RNA was quantified by a NanoDrop ND-2000 (Thermo Scientific, SA, USA), and RNA integrity was assessed using an Agilent Bioanalyzer 2100 (Agilent Technologies). Sample labeling, microarray hybridization, and washing were performed according to the manufacturer’s protocols. Briefly, total RNA was dephosphorylated, denatured, and then labeled with cyanine-3-CTP. After purification, the labeled RNAs were hybridized into the microarray. After being washed, the arrays were scanned with an Agilent Scanner G2505C (Agilent Technologies). Feature Extraction software (version 10.7.1.1, Agilent Technologies) was used to analyze the array of images to obtain raw data. Next, Genespring software (version 14.8, Agilent Technologies) was utilized to finish the basic analysis of the raw data. First, the raw data were normalized with the quantile algorithm. The probes that had at least 100.0 percent of samples in any 1 condition out of 2 conditions with flags in “Detected” were chosen for further data analysis. Differentially expressed miRNAs and mRNAs were then identified using R software (Version 3.6.1) with the “limma” package through fold change (FC) and adjusted *P* value. The threshold for up- and down-regulated genes was set at an FC >4.0 and an adjusted *P* value <0.05. The differentially expressed miRNAs were further validated by quantitative reverse transcriptase–polymerase chain reaction (qRT-PCR). Target genes of the candidate miRNA were identified based on the intersection between differentially expressed mRNAs and different predicted algorithms (Funrich software and five online predicting databases, including miRDB, miRanda, TargetScan, PITA, and PicTar). Gene ontology (GO) analysis and Kyoto Encyclopedia of Genes and Genomes (KEGG) analysis were applied to determine the roles of these target genes by R. Hierarchical clustering were performed to show the differential miRNA and mRNA expression patterns among samples using scatter plots, volcano plots and heatmaps for visualization.

### Western blotting

As described by previous studies, western blotting analysis was performed to assess expressions of proteins^55^. Briefly, protein lysates were prepared from BMSCs or EVs using RIPA lysis buffer containing phosphatase inhibitors and protease. The protein concentrations determination was performed using a BCA Protein Assay Reagent Kit (Pierce Biotechnology, Rockford, IL, USA).

Equivalent amounts of proteins (15 μg) were resolved by sodium dodecyl sulfate-polyacrylamide gel electrophoresis (SDS-PAGE) and then transferred onto the polyvinylidene fluoride (PVDF) membrane. After being blocked by 5% non-fat dry milk in Tris-buffered saline for 1 h (room temperature), the membranes were incubated with primary antibodies. After incubation with horseradish peroxidase (HRP)-conjugated species-matched secondary antibodies, the blots were then visualized using chemiluminescence kits (Amersham Corp, Buckinghamshire, UK). The densitometric analysis of blots was performed using ImageJ software (Media Cybernetics, Silver Spring, MD, USA). β-actin (ab8227, Abcam, dilution at 1:2000) was utilized as the normal control. All antibodies used have been listed in Supplementary Table 1. All blots from the same experiment were processed in parallel. Uncropped blots are shown in Supplementary Fig. 8.

#### RNA isolation, cDNA synthesis, and qRT-PCR

Total RNA isolation from joint tissues or BMSCs was performed using TRIzol reagent (Invitrogen, Carlsbad, CA, USA) according to the manufacturer’s instructions. RNA quality and quantity determination were performed using a bioanalyzer (Agilent Inc., Santa Clara, CA, USA) and nanodrop (Thermo Scientific). For quantitative detection of miRNA and mRNA, RT-PCR was performed using a qSYBR-green-containing PCR kit (Qiagen, Germantown, MD, USA) with an RT-PCR system (Applied Biosystems, SA, USA). U6 small nuclear RNA (snRNA) and GAPDH were used as the housekeeping gene for normalization. mRNA and miRNA qRT-PCR primers and internal control were purchased from Applied Biosystems. All primers have been listed in Supplementary Table 2. All PCR assays were performed in triplicate using the 2^−△△Ct^ method. All Primer sequences are available from the authors upon request.

#### In vitro miRNA and siRNA transfection

Human BMSCs were transfected with miRNA-455 mimics, inhibitors, or negative control (customized and purchased from RIBOBIO, Guangzhou, China) using Lipofectamine RNAiMAX transfection reagent (Invitrogen). Cells were transfected with SOX11 siRNA, FOXO1 siRNA, or control siRNA (Thermo Scientific) using Lipofectamine 3000 (Invitrogen) according to the manufacturer’s instructions. The SOX11 expression plasmid was obtained using the pcDNA™ 3.1/V5-His TOPO™ TA Expression Kit (Invitrogen™). After 48 h of transfection, the expression levels of the target genes were evaluated from the collected cellular lysates by qRT-PCR and western blotting of specific genes in the miR-455/SOX11/FOXO signaling axis (ab185966, ab232628, ab34712, ab39670, ab17091, ab39012, ab32034, ab200738, and ab180768, Abcam, dilution at 1:1000–2000).

#### 3′-Untranslated region (UTR) cloning and luciferase assay

To construct the SOX11 3′-UTR-Luc reporter plasmid, a wild-type (Wt) or mutant (Mut) fragment of the 3′-UTR of SOX11 containing the predicted miR-455 binding sites was PCR-amplified and inserted into the psi-CHECKTM-2 vector (Promega, Madison, WI). To conduct the luciferase assay, human BMSCs were seeded into a 96-well plate and co-transfected with WT- or Mut-SOX11 3′-UTR-Luc reporter plasmids and a miR-455 mimic, inhibitor, or negative control with Lipofectamine PLUSTM reagent (Invitrogen, China). At 48 h after transfection, the cell lysates were harvested for luciferase activity determination using the Dual-Glo Luciferase Assay system (Promega, Madison, WI, USA) and were normalized to firefly luciferase activity. Each experiment was performed in triplicate and independently replicated three times.

#### Cell immunofluorescence

Cell immunofluorescence analysis was performed according to the descriptions by the previous studies^56^. Briefly, cells in plates were fixed in 4% PFA, permeabilized with PBS containing 0.5% Triton X-100 for 20 min, and then blocked with 3% BSA containing 0.025% Triton X-100 and 5% FBS at room temperature for 30 min. Cells were immunostained via incubation with primary antibodies at 4 °C overnight. After that, the cells were washed using PBS three times and incubated with appropriate secondary antibodies. All secondary antibodies were listed in Supplementary Table 1. After the cells were washed, DAPI was used for nuclear counterstaining for 5 min. Immunofluorescence visualization was performed using a confocal microscope (Carl Zeiss, Germany). Each experiment was conducted in triplicate, and representative confocal microscopy images are shown.

#### Fluorescence in situ hybridization (FISH)

FISH experiments were performed for cell samples from BMSCs, and cartilage tissues from animal experiments. The frozen sample tissues were fixed in 4% PFA for 10 min and washed with PBS 3 times (5 min each time). After being digested in 20 µg/ml protease K for 15 min, the slides were prehybridized for 1 h at 37 °C and then incubated in an 8 ng/µl Cy3-conjugated hsa-miR-455 probe (synthesized by Genscript, China) directed against the full length mature miR-455 sequence in the hybridization mixture at 37 °C overnight. Then, the slides were washed using 2× SSC (10 min) at 37 °C, 1× SSC (2 × 5 min) at 37 °C, and 0.5× SSC (10 min) at RT. The sections were then counterstained with DAPI and incubated for 8 min. The slides were observed with a NIKON biological microscope (Nikon Eclipse ci, Nikon Corporation, Tokyo, Japan) and imaged using an imaging system (Nikon DS-U3, Nikon Corporation, Tokyo, Japan). All the experimental procedures were conducted in triplicate within an RNase-free environment after DEPC processing.

### Animal experiments

#### Cartilage repair with T3-EV gel in vivo

Collected sEVs (10 × 10^8^ particles/ml) and BMSC suspension (a total of 1 × 106 cells/ml) was loaded into the composite hydrogel^7^ as previously described. (Supplementary Table 3) Composite hydrogel as the sEVs and BMSC carrier material is a mixture of gelatin, fibrinogen, HA, and glycerol (all purchased from Sigma-Aldrich). In brief, HA was dissolved in DMEM (high glucose) by stirring the solution at 37 °C overnight. Glycerol was added to the solution and stirred for 1 h, which was further shaken after adding gelatin and fibrinogen for 1 h and resulted in final concentrations of gelatin (45 mg/ml), fibrinogen (30 mg/ml), HA (3 mg/ml), and glycerol (10% v/v). The prepared solution was filtered through a 0.45-μm syringe filter and was stored at −20 °C before use. The animal experiment protocols were approved by Shanghai Ninth Hospital, the medical school of Shanghai Jiao Tong University Ethics Committee, the local Institutional Animal Care and Use Committee (IACUC), and complied with the Guide for the Care and Use of Laboratory Animals published by the National Academy Press (National Institutes of Health Publication No. 85-23, revised 1996). Adult male rats were used for the cartilage injury model. Joint cartilage injury was performed (*n* = 6) as reported^57^. Briefly, an incision was made to open up the skin over the rat knee joint area, followed by an incision along the medial side of the patellar ligament and through the quadriceps muscle to aid patellar dislocation. The patellar groove was exposed and a 3 mm × 3 mm cylindrical full-thickness cartilage defect was created at the non-weight-bearing surface along the length of the groove using an electric drill. Before implantation, the composite hydrogel was cross-linked by the addition of a thrombin solution (20 UI/ml, Sigma-Aldrich) for 30 min at room temperature. Then the uncross-linked components (gelatin, HA, and glycerol) were washed out with PBS solution three times. After cross-linking, the T3-EV-embedded composite hydrogel or un-EV hydrogel was injected into the defect site for cartilage repair in different groups. The patella was then relocated, and the joint capsule and skin were sutured separately. The contralateral leg served as the internal uninjured control. Rats were sacrificed after 24 weeks for further study. Cartilage samples were fixed in 4% paraformaldehyde, processed, and embedded in paraffin. Serial sections of the cartilage were stained with HE and safranin-O according to standard protocols. Immunohistochemical staining of SOX9, ACAN, MMP13, and other markers in the miR-455/SOX11/ FOXO signaling axis (ab185966, ab232628, ab39670, ab170916, and ab39012, Abcam, dilution at 1:1000) was conducted according to standard protocols for the repaired cartilage sections in different groups. The stained images were taken using a light microscope. A modified method was used to evaluate the histological repair of articular cartilage defects^58^. The chondroprotective effects of the scaffolds were examined by evaluating the cartilages of the medial FC and TP according to the criteria of the ICRS cartilage lesion classification system and Mankin grading system^59^.

#### Injection of T3-EV for OA treatment in the rat knee joint

Rats were used to examine the effect of T3-BMSC-EV in an OA model in vivo. After the skin incision, a 3-cm medial parapatellar incision was applied and the patella was dislocated. Anterior cruciate ligament transection was performed to construct an OA model. After the operation, rats were allowed to move freely in their single cages and fed with standard food and water. Rats were randomized into three groups (*n* = 8 for each group; two knees of each rat were used): the Saline group with intra-articular saline injection, the un-EV group with untreated-BMSC-EV injection, and the T3-EV group with TGFβ3-treated BMSC-derived EV injection. On the first day of every week from the fifth to the twelfth week after surgery, rats in the un-EV and T3-EV treatment groups received intra-articular injection with 50 μL un-EVs (10^10^ particles/mL) or 50 μL T3-EVs (10^10^ particles/mL) in PBS, respectively. Rats in the saline group were injected intra-articularly with 50 μL saline at each time point. Serial sections (4-μm thick) were cut sagittally through the center of the most diseased osteoarthritic site and stained with H&E and Toluidine blue according to standard protocols. Immunohistochemical staining of cartilage marker and markers in the miR-455/SOX11/ FOXO signaling axis (ab185966, ab232628, ab39670, ab170916, and ab39012, Abcam, dilution at 1:1000) were also conducted according to standard protocols in the generated cartilage tissue sections in different groups compared to the native cartilage. The stained images were taken using a light microscope. Histological assessment of sagittal sections of the knee joints was conducted by two blinded observers who followed the Osteoarthritis Research Society International (OARSI) scoring system^60^. Measurements were also performed for osteophyte maturation, synovitis score (0–3, 0 = no synovial thickening; 1 = lining of two cell layers; 2 = several extra cell layers; 3 = clear inflammation with cell infiltrate or exudate), and subchondral bone plate thickness (the region between the osteochondral junction and marrow space on the medial side of the tibial plateau) using Bioquant Osteo software (BIOQUANT, Inc.) as describe previously^61,62^.

#### Immunofluorescence staining of histological sections

To evaluate the distribution and expression of proteins, immunofluorescence analysis was performed as described by the previous reports^63^. In brief, frozen sections of collected tissues with a thickness of 6 μm were fixed in 1% paraformaldehyde and washed using PBS. After blocking with 5% normal goat serum diluted in PBS, sections were then incubated with primary antibodies in PBS with 1% goat serum (4 °C, overnight). After washing with PBS, sections were incubated with secondary antibodies for 1 h. Images visualization was performed using confocal microscopy (Olympus, Tokyo, Japan). The primary antibodies in this study were available upon reasonable request.

#### Statistical analysis

The software of SPSS (Version 19.0, SPSS Inc., Chicago, IL, USA) and GraphPad Prism (Version 8.0, GraphPad Software Inc., San Diego, CA, USA) were used for statistical analysis. Mann–Whitney *U*-test, Student’s *t*-test, and one-way ANOVA test were used for data analysis as appropriate. *P* < 0.05 was considered statistically significant.

### Reporting summary

Further information on research design is available in the Nature Research Reporting Summary linked to this article.

## Supplementary information

supplementary Material

REPORTING SUMMARY

## Data Availability

All data needed to evaluate or reproduce the conclusions in the paper are present in the paper and/or the Supplementary Materials.
