# Supplementary material for: Chondrogenic primed extracellular vesicles activate miR-455/SOX11/FOXO axis for cartilage regeneration and osteoarthritis treatment
Source: NPJ Regen Med. 2022 Sep 16;7:53. doi: 10.1038/s41536-022-00250-7 (PMC9481593; doi:10.1038/s41536-022-00250-7)
Supplement: Supplementary file 1 — supplementary Material [file 41536_2022_250_MOESM1_ESM.pdf]

Supplementary Information

Supplementary Table 1. List of all antibodies used in the experiment

Antibodies	manufacturers	Catalog Number	species origins	final dilutions
ALIX	Abcam	ab275377	Rabbit	1:1000
CD9	Abcam	ab236630	Rabbit	1:1000
CD63	Abcam	ab134045	Rabbit	1:1000
CD81	Abcam	ab109201	Rabbit	1:1000
SOX9	Abcam	ab185966	Rabbit	1:1000
ACAN	Abcam	ab232628	Rabbit	1:1000
COL2A1	Abcam	ab34712	Rabbit	1:2000
FOXO1	Abcam	ab39670	Rabbit	1:1000
SOX11	Abcam	ab170916	Rabbit	1:1000
MMP13	Abcam	ab39012	Rabbit	1:1000
p27	Abcam	ab32034	Rabbit	1:1000
Cathepsin L	Abcam	ab200738	Rabbit	1:1000
Gadd45a	Abcam	ab180768	Rabbit	1:1000
β-actin	Abcam	ab8227	Rabbit	1:2000

Goat Anti-Rabbit IgG H&L (Alexa Fluor® 488)	Abcam	ab150077	Goat	1:500
Goat Anti-Rabbit IgG H&L (HRP)	Abcam	ab6721	Goat	1:1000
Goat Anti-Rabbit IgG H&L (Alexa Fluor® 647)	Abcam	ab150079	Goat	1:500
FITC Anti-CD29	Abcam	ab150002	Mouse	1:2000
FITC Anti-CD34	Abcam	ab78165	Mouse	1:2000
FITC Anti-CD45	Abcam	ab27287	Mouse	1:2000
FITC Anti-CD90	Abcam	ab124527	Mouse	1:2000
FITC Anti-CD105	Abcam	ab11415	Mouse	1:2000

Supplementary Table 2. RT-qPCR primers for amplifying the gene makers.

Gene name	Forward primer (5'->3')	Reverse primer (5'->3')
SOX9	GGCAAGCTCTGGAGACTTCTG	CCCGTTCTTCACCGACTTCC
ACAN	AGTGCACAGAGGGGTTTGTC	CGTTTGTAGGTGGTGGGGTC
COL2A1	GGGATCGTGGTGACAAAGGT	CTGGGCAGCAAAGTTTCCAC
MMP13	TCCAGTCTCTCTATGGTCCAGG	CCTCGGAGACTGGTAATGGC
FOXO1	GAGGGTTAGTGAGCAGGTTACA	ACTGCTTCTCTCAGTTCCTGC
Gadd45a	AGAAGACCGAAAGCGACCC	GTTGATGTCGTTCTCGCAGC
p27	CTGAACGGAGCTGAAGTCG	TAACCGCGCAGCAGATAGT
Cathepsin L	AGAGCGTCTACCCCGAACT	CAGAGCTGTAGGAGCTGTGTC
SOX11	CCTGTCGCTGGTGGATAAGG	GTGCAGTAGTCGGGGAACTC
ADAMTS4	CCTTCAGGAAATTCAGGTACGG	CCAAGTAGATGCTCCGGTGG
ADAMTS5	TGGCTCACGAAATCGGACATT	GCATTTGGACCAGGGCTTAG
MMP9	GCGGAGATTGGGAACCAG	TTGTCGGCGATAAGGAAGG
MMP14	GAGCATTCCAGTGACCCCTC	ACCCTGACTCACCCCCATAA
TIMP1	ATTCCGACCTCGTCATCAGG	GCATCCCCTAAGGCTTGGAA
TIMP3	ACCGAGGCTTCACCAAGATG	CCATCATAGACGCGACCTGT
FABP4	AAACTGGTGGTGGAATGCGT	GCGAACTTCAGTCCAGGTCA
CEBPB	TGACGCAGCGGTTGCTA	CGGCTCTGACTCGCTAAAGT
OCN	CACCGAGACACCATGAGAGC	CTGCTTGGACACAAAGGCTGC
RUNX2	TCTCCAGGAGGACAGCAAGA	CTGCTTGCAGCCTTAAATGACT
Nanog	TACCTCAGCCTCCAGCAGAT	ACCAGGTCTTCACCTGTTTGT
Oct4	GGAAAAGCAACTGCCTCCCT	ATGCTGCCCTTGTGGATGTC
GAPDH	GGACCTGACCTGCCGTCTAG	GTAGCCCAGGATGCCCTTGA
miR-455-5p	CGCGTATGTGCCTTTGGACT	GTCGTATCCAGTGCAGGGTC
RT-Primer	GTCGTATCCAGTGCAGGGTCCGAGGTATTCGCACTGGATACGACCGATGT	
miR-146a-5p	CGCGTGAGAACTGAATTCCATG	GTCGTATCCAGTGCAGGGTC
RT-Primer	GTCGTATCCAGTGCAGGGTCCGAGGTATTCGCACTGGATACGACAACCCA	
miR-557	GATGTGTTTGCACGGGTGG	GTCGTATCCAGTGCAGGGTC
RT-Primer	GTCGTATCCAGTGCAGGGTCCGAGGTATTCGCACTGGATACGACAGACAA	
miR-146b-5p	GCGTGAGAACTGAATTCCATAGG	GTCGTATCCAGTGCAGGGTC
RT-Primer	GTCGTATCCAGTGCAGGGTCCGAGGTATTCGCACTGGATACGACCAGCCT	
miR-126-5p	CGCGCATTATTACTTTTGGTACG	GTCGTATCCAGTGCAGGGTC
RT-Primer	GTCGTATCCAGTGCAGGGTCCGAGGTATTCGCACTGGATACGACCGCGTA	

Supplementary Table 3.The recipe of generated T3-EV composite hydrogel for cartilage repair

	EV concentration	Gelatin	Fibrinogen	HA	Glycerol	BMSC density
**T3-EV hydrogel**	T3-EV (10x10^8 particles/ml)	45 mg/ml	30 mg/ml	3 mg/ml	10% v/v	1×10^6/ml
**un-EV Hydrogel**	Untreated-EV (10x10^8 particles/ml)	45 mg/ml	30 mg/ml	3 mg/ml	10% v/v	1×10^6/ml
**Control**	None	45 mg/ml	30 mg/ml	3 mg/ml	10% v/v	1×10^6/ml

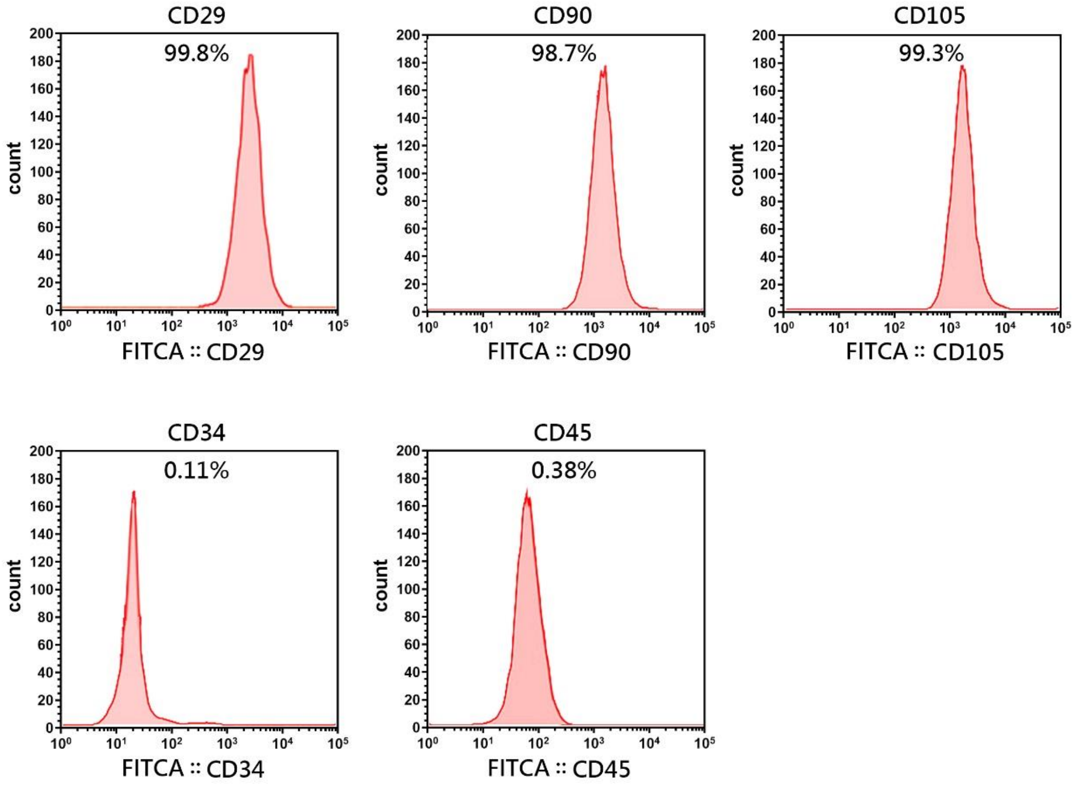

Supplementary Figure 1. Characterization of the isolated BMSCs. BMSCs surface markers (CD29, CD34, CD45, CD90 and CD105) were assessed by flow cytometry.

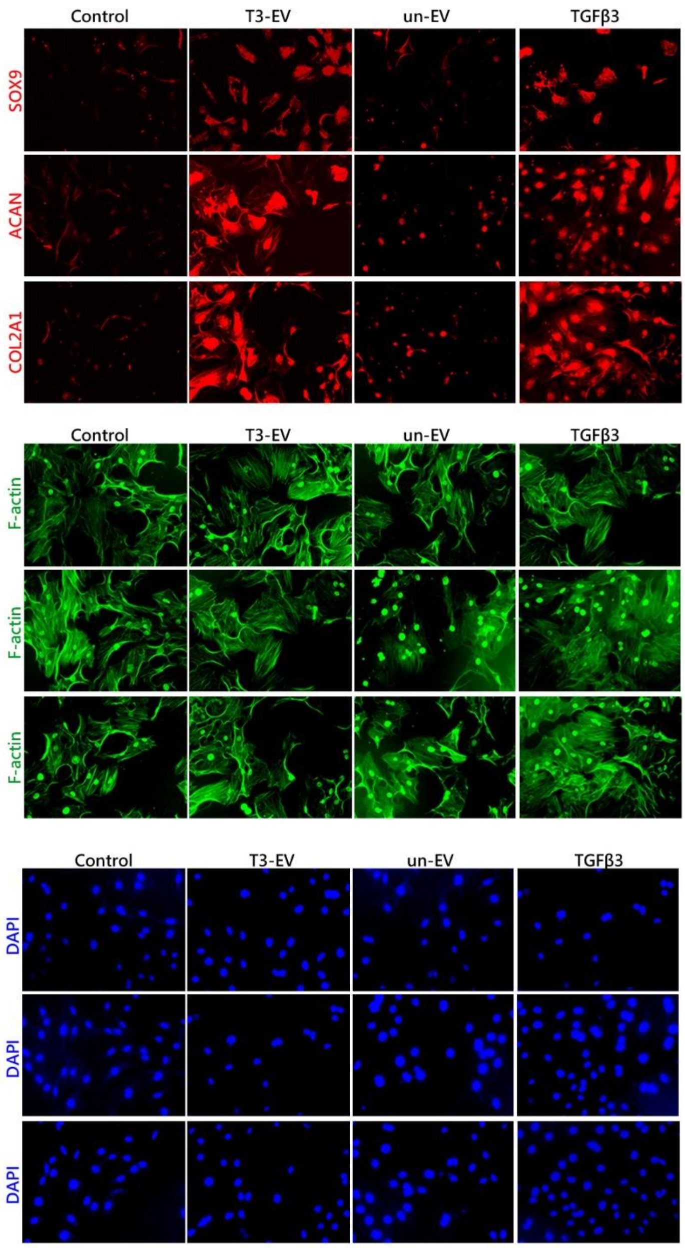

Supplementary Figure 2. Each channel presented for Figure 1G

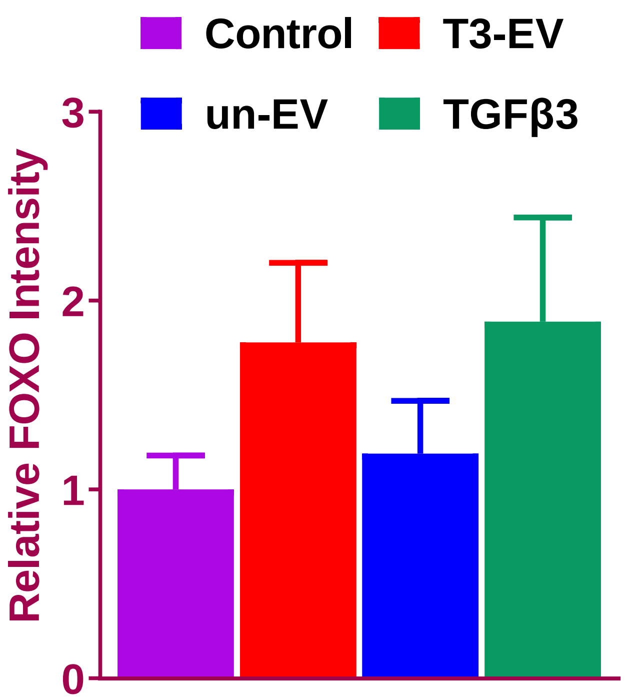

Supplementary Figure 3. Levels of protein expression as determined by western blot in Figure 3F. The graph shows different protein level relative to the level of the control in Figure 3F; n=3.

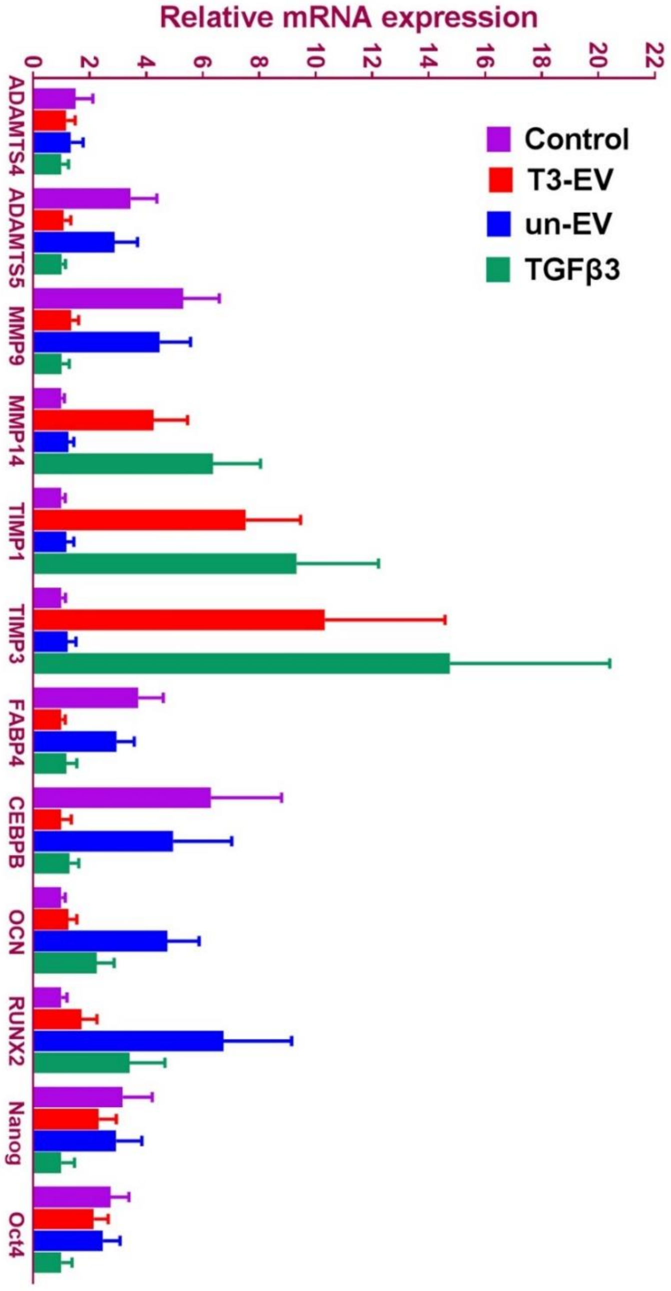

Supplementary Figure 4. Quantification of gene expression with the same BMSCs and the same EVs (n=3 for each) with qRT-PCR for ECM remodeling genes（ADAMTS4, ADAMTS5, MMP9, MMP14, TIMP1, TIMP3） adopogenesis-related genes (FABP4, CEBPB), osteogenesis-related genes (OCN, RUNX2) and pluripotency genes (Nanog, Oct4)

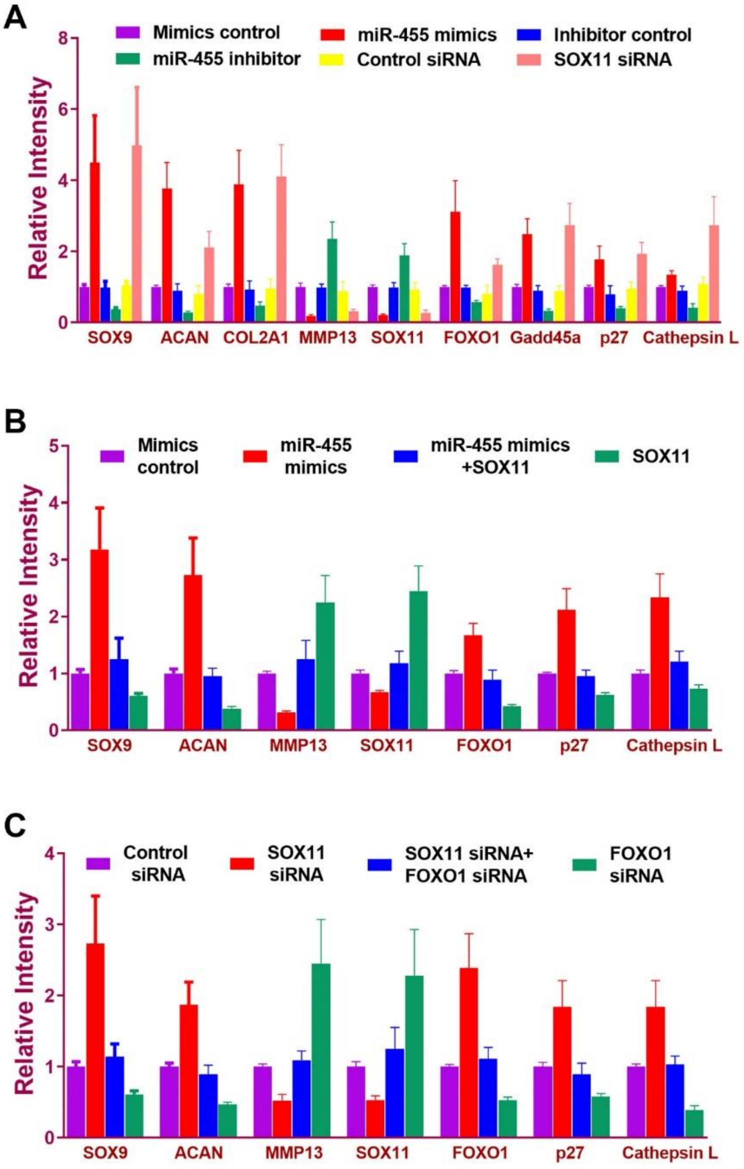

Supplementary Figure 5. Levels of protein expression as determined by western blot in Figure 5C-E. A. The graph shows different protein level relative to the level by treatment with mimics control in **Figure 5C**; n=3. **B.** The graph shows different protein level relative to the level by treatment with mimics control in **Figure 5D**; n=3. **C.** The graph shows different protein level relative to the level by treatment with control siRNA in **Figure 5E**; n=3.

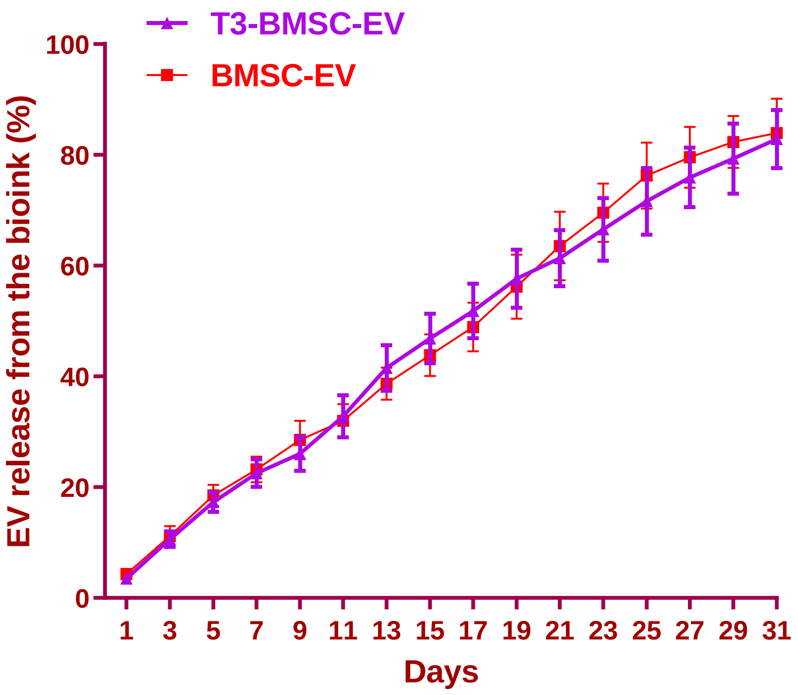

Supplementary Figure 6. EV release rate from the hydrogel bioink in a month.

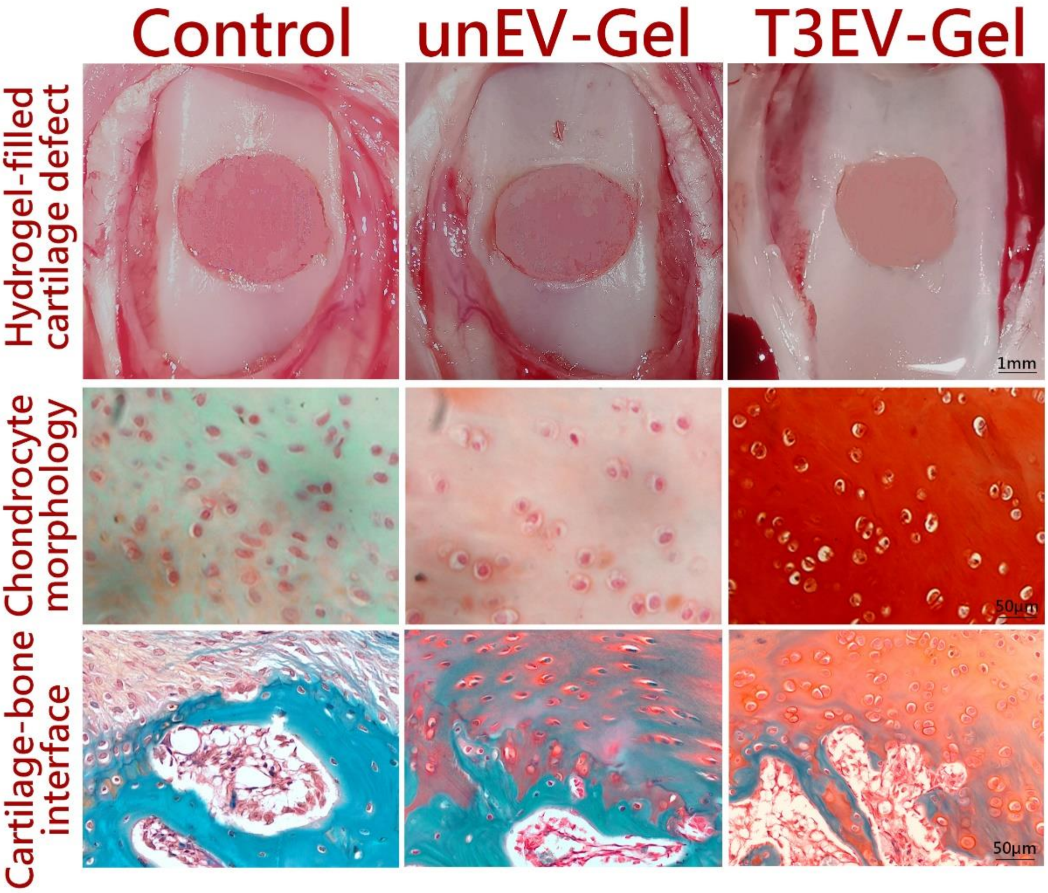

Supplementary Figure 7. Higher-resolution of chondrocyte morphology and cartilage-bone interface in the cartilage repair model. **1st row**: Gross appearance of hydrogel-filled cartilage defect at baseline. **2nd row**: Higher-resolution of chondrocyte morphology in the repaired cartilage; **3rd row**: Cartilage-bone interface in different groups in the repaired tissues.

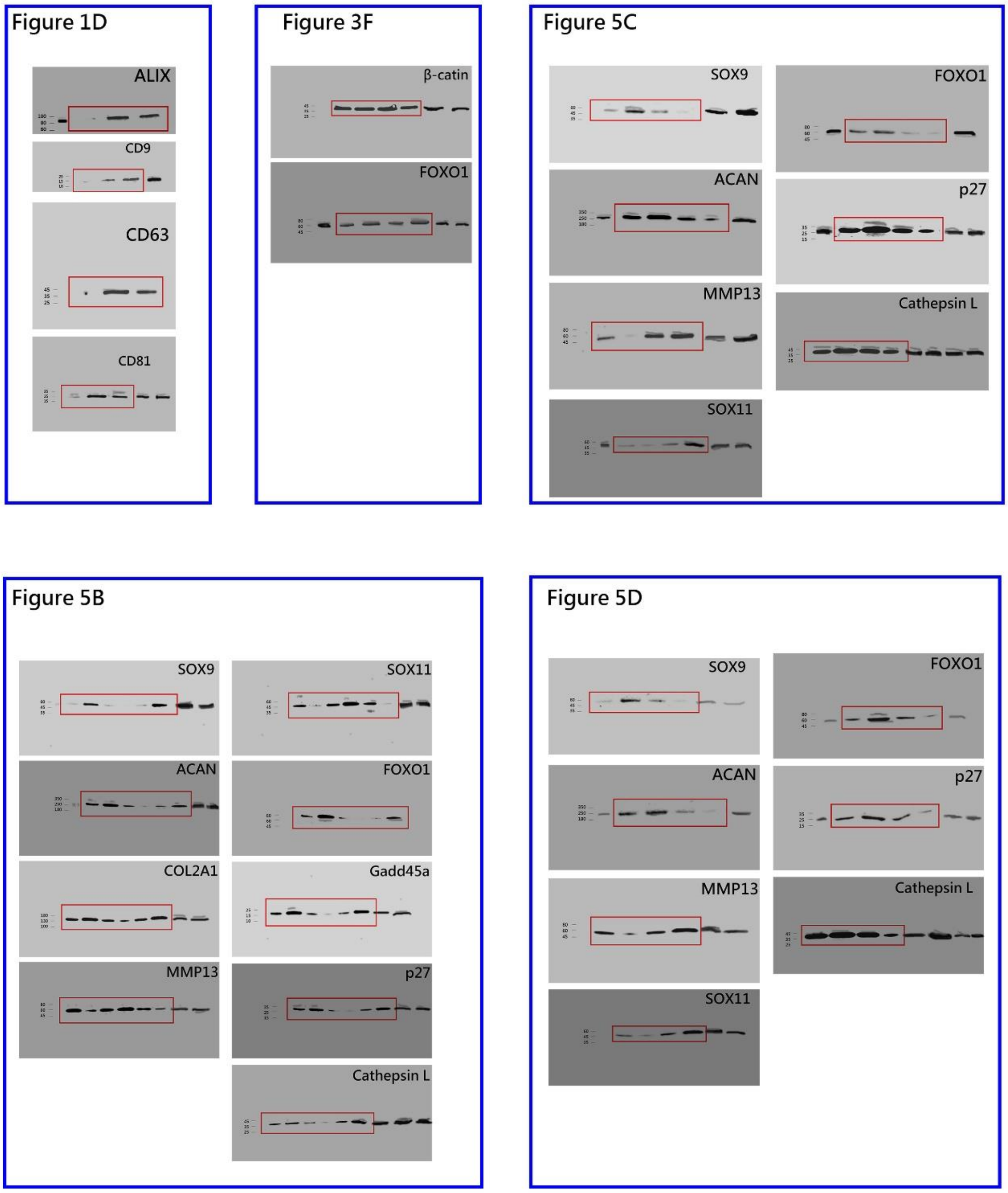

Supplementary Figure 8. Uncropped blots relate to figures in this study. Red square frames indicate the representative bands used in the main text.
