# Supplementary material for: Chondrogenic primed extracellular vesicles activate miR-455/SOX11/FOXO axis for cartilage regeneration and osteoarthritis treatment
Source: NPJ Regen Med. 2022 Sep 16;7:53. doi: 10.1038/s41536-022-00250-7 (PMC9481593; doi:10.1038/s41536-022-00250-7)
Supplement: Supplementary file 2 — REPORTING SUMMARY [file 41536_2022_250_MOESM2_ESM.pdf]

nature portfolio

Corresponding author(s): sun ye

Last updated by author(s): Jun 30, 2022

Reporting Summary

Nature Portfolio wishes to improve the reproducibility of the work that we publish. This form provides structure for consistency and transparency in reporting. For further information on Nature Portfolio policies, see our Editorial Policies and the Editorial Policy Checklist.

Statistics

For all statistical analyses, confirm that the following items are present in the figure legend, table legend, main text, or Methods section.

n/a	Confirmed	
☐	☒	The exact sample size (*n*) for each experimental group/condition, given as a discrete number and unit of measurement
☐	☒	A statement on whether measurements were taken from distinct samples or whether the same sample was measured repeatedly
☐	☒	The statistical test(s) used AND whether they are one- or two-sided<br>*Only common tests should be described solely by name; describe more complex techniques in the Methods section.*
☐	☒	A description of all covariates tested
☐	☒	A description of any assumptions or corrections, such as tests of normality and adjustment for multiple comparisons
☐	☒	A full description of the statistical parameters including central tendency (e.g. means) or other basic estimates (e.g. regression coefficient) AND variation (e.g. standard deviation) or associated estimates of uncertainty (e.g. confidence intervals)
☐	☒	For null hypothesis testing, the test statistic (e.g. *F*, *t*, *r*) with confidence intervals, effect sizes, degrees of freedom and *P* value noted<br>*Give P values as exact values whenever suitable.*
☐	☒	For Bayesian analysis, information on the choice of priors and Markov chain Monte Carlo settings
☐	☒	For hierarchical and complex designs, identification of the appropriate level for tests and full reporting of outcomes
☐	☒	Estimates of effect sizes (e.g. Cohen's *d*, Pearson's *r*), indicating how they were calculated

Our web collection on statistics for biologists contains articles on many of the points above.

Software and code

Policy information about availability of computer code

Data collection	The software of SPSS (Version 19.0, SPSS Inc., Chicago, IL, USA) and GraphPad Prism (Version 8.0, GraphPad Software Inc., San Diego, CA, USA) were used for statistical analysis.
Data analysis	The software of SPSS (Version 19.0, SPSS Inc., Chicago, IL, USA) and GraphPad Prism (Version 8.0, GraphPad Software Inc., San Diego, CA, USA) were used for statistical analysis.

For manuscripts utilizing custom algorithms or software that are central to the research but not yet described in published literature, software must be made available to editors and reviewers. We strongly encourage code deposition in a community repository (e.g. GitHub). See the Nature Portfolio guidelines for submitting code & software for further information.

Data

Policy information about availability of data

All manuscripts must include a data availability statement. This statement should provide the following information, where applicable:

- Accession codes, unique identifiers, or web links for publicly available datasets
- A description of any restrictions on data availability
- For clinical datasets or third party data, please ensure that the statement adheres to our policy

All data needed to evaluate the conclusions in the paper are present in the paper and/or the Supplementary Materials.

Human research participants

Policy information about studies involving human research participants and Sex and Gender in Research.

Reporting on sex and gender	Sex and gender was not considered in the study.
Population characteristics	Human BMSCs were isolated from biopsies of healthy clinical donors.
Recruitment	It was provided in the supplementary files.
Ethics oversight	The ethical committee for shanghai ninth hospital (school of medicine, Shanghai jiaotong university) approved all procedures, and patients' informed consent forms were obtained.

Note that full information on the approval of the study protocol must also be provided in the manuscript.

Field-specific reporting

Please select the one below that is the best fit for your research. If you are not sure, read the appropriate sections before making your selection.

☒ Life sciences ☐ Behavioural & social sciences ☐ Ecological, evolutionary & environmental sciences

For a reference copy of the document with all sections, see nature.com/documents/nr-reporting-summary-flat.pdf

Life sciences study design

All studies must disclose on these points even when the disclosure is negative.

Sample size	It was provided in the supplementary files.
Data exclusions	It was provided in the supplementary files.
Replication	Experiments were all reproduced three times.
Randomization	Randomization is not relevant in our study since we do not have to categorize the patients into groups.
Blinding	Blinding of different groups was made for those who performed the experiment

Reporting for specific materials, systems and methods

We require information from authors about some types of materials, experimental systems and methods used in many studies. Here, indicate whether each material, system or method listed is relevant to your study. If you are not sure if a list item applies to your research, read the appropriate section before selecting a response.

Materials & experimental systems

n/a	Involved in the study	
☐	☒	Antibodies
☐	☒	Eukaryotic cell lines
☒	☐	Palaeontology and archaeology
☐	☒	Animals and other organisms
☒	☐	Clinical data
☒	☐	Dual use research of concern

Methods

n/a	Involved in the study	
☒	☐	ChIP-seq
☐	☒	Flow cytometry
☒	☐	MRI-based neuroimaging

Antibodies

Antibodies used	Antibodies were provided in the supplementary files.
Validation	Antibodies and their validation were provided in the supplementary files.

Eukaryotic cell lines

Policy information about cell lines and Sex and Gender in Research

Cell line source(s)	Cell line source was provided in the supplementary files.
Authentication	Cell line source was authenticated with flow cytometry in the supplementary files.
Mycoplasma contamination	Cell line source was not tested for mycoplasma contamination.
Commonly misidentified lines (See ICLAC register)	Not relevant. Cell source was provided in the manuscript and the supplementary files.

Animals and other research organisms

Policy information about studies involving animals; ARRIVE guidelines recommended for reporting animal research, and Sex and Gender in Research

Laboratory animals	It was provided in the supplementary files.
Wild animals	No relevant.
Reporting on sex	*Indicate if findings apply to only one sex; describe whether sex was considered in study design, methods used for assigning sex. Provide data disaggregated for sex where this information has been collected in the source data as appropriate; provide overall numbers in this Reporting Summary. Please state if this information has not been collected. Report sex-based analyses where performed, justify reasons for lack of sex-based analysis.*
Field-collected samples	It was provided in the supplementary files.
Ethics oversight	It was provided in the supplementary files.

Note that full information on the approval of the study protocol must also be provided in the manuscript.

Flow Cytometry

Plots

Confirm that:

☒ The axis labels state the marker and fluorochrome used (e.g. CD4-FITC).

☒ The axis scales are clearly visible. Include numbers along axes only for bottom left plot of group (a 'group' is an analysis of identical markers).

☒ All plots are contour plots with outliers or pseudocolor plots.

☒ A numerical value for number of cells or percentage (with statistics) is provided.

Methodology

Sample preparation	It was provided in the supplementary files.
Instrument	It was provided in the supplementary files.
Software	It was provided in the supplementary files.
Cell population abundance	It was provided in the supplementary files.
Gating strategy	It was provided in the supplementary files.

☒ Tick this box to confirm that a figure exemplifying the gating strategy is provided in the Supplementary Information.
